# Supplementary material for: Photoredox phase engineering of transition metal dichalcogenides
Source: Nature. 2024 Aug 28;633(8028):83–9. doi: 10.1038/s41586-024-07872-5 (PMC11374681; doi:10.1038/s41586-024-07872-5)
Supplement: Supplementary file 1 — Supplementary Information [file 41586_2024_7872_MOESM1_ESM.docx]

Supplementary Information for

**Photo-Redox Phase Engineering of Transition Metal Dichalcogenides**

Juhwan Lim^1,2^, Jung-In Lee^2^, Ye Wang^2^, Nicolas Gauriot^1^, Ebin Sebastian^1^, Manish Chhowalla^2^, Christoph Schnedermann^1†^, & Akshay Rao^1†^

^1^Cavendish Laboratory, University of Cambridge, Cambridge, UK.

^2^Department of Materials Science & Metallurgy, University of Cambridge, Cambridge, UK.

**1. Wavelength dependent Diffraction-limited optical Imaging of TMDs**

The TMD flakes observed under blue (455 nm) will appear sharper compared to red (730 nm) illumination due to the difference in diffraction limit, which can be estimated by the Abbe formula: d = λ/(2NA). Here, d is the size of a feature that can be truthfully represented, λ is the wavelength and NA the numerical aperture of the objective (1.4 in our case). At 730 nm, d_730_ = 260 nm, while at 455 nm, d_455_ = 162 nm.


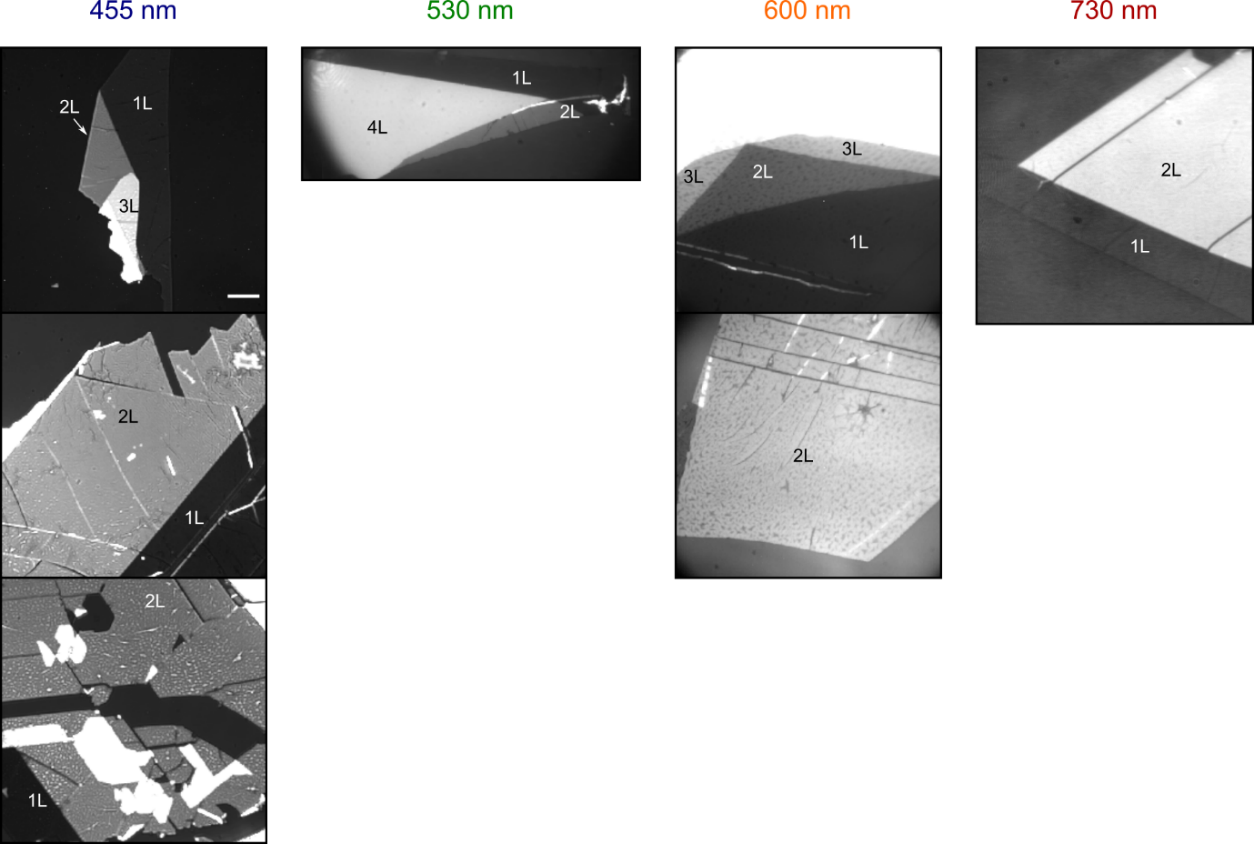


**Fig. S1. Optical image of MoS_2_ using different wavelengths. Scale bar 5 µm (applies to all images).**

Therefore 455 nm imaging resolves more high-frequency components and is more sensitive to the morphology of the flake and therefore the preparation procedure. Since the optical image represents to first approximation a convolution of the actual surface roughness with a Gaussian point spread function of full width half max = d^1^. This is also found in Fig. S1.

In Fig. 2, a rough surface will therefore appear significantly smoother when inspected at 730 nm compared to 455 nm. Given that the flakes in our experiments are mechanically exfoliated and not perfectly smooth, we expect generally a broader intensity histogram at 455 nm compared to 730 nm. Both the 2H and 1T phases are sufficiently different that they exhibit different optical responses at different wavelengths. This manifests primarily in a different degree of intensity loss during the phase transition, which we observed in Fig. 2. Due to the normalisation procedure, we would expect that the histogram narrows by the same fraction as the optical intensity of the flake reduces during the reaction.


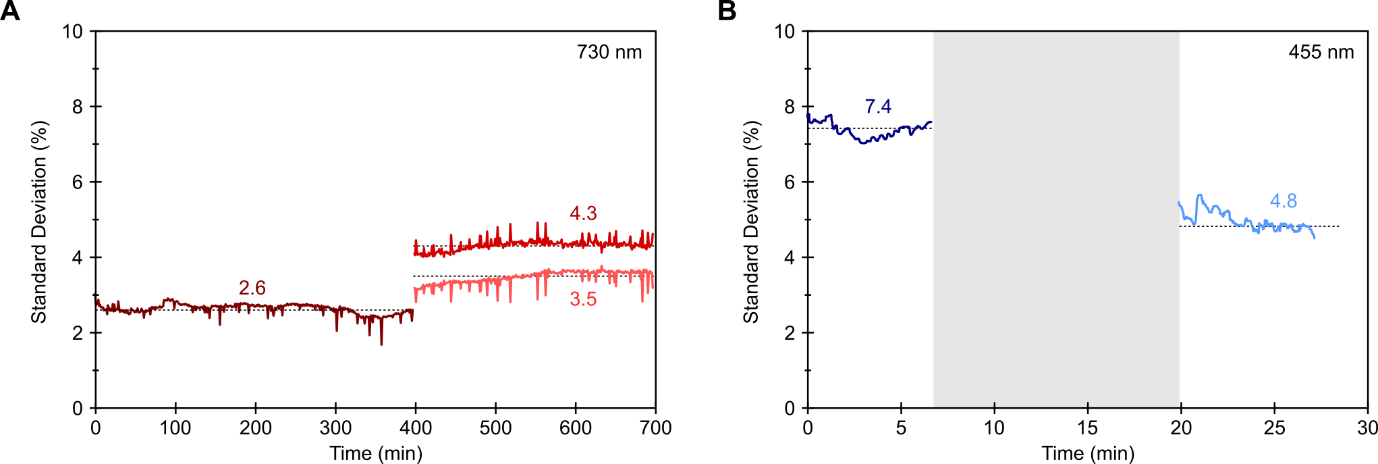


**Fig. S2. Histogram fit analysis of bilayer data represented in Fig. 2.** **A,B** standard deviation retrieved from a Gaussian fit to the histogram maps shown in Fig. 2 for 730 and 455 nm, respectively. For 730 nm, the 2H distribution is fitted to a single Gaussian function until 400 min, after which the 1T bi-peaked phase was visually established and fit to a sum of two Gaussian functions. For 450 nm, we only fitted the beginning (2H) and end (1T) state to a single Gaussian function, since the fast peak broadening and shifting during the transition (grey box) cannot be accurately described with a single Gaussian.

To further quantify the initial heterogeneity, we have further analysed our experimental data and examined the width of the intensity histogram throughout the reaction. By fitting the intensity histogram to a Gaussian and taking into account the diffraction limit, we can qualify the heterogeneity between the different flakes.

In Fig. S2, we find a significantly narrower distribution for initial bilayer 2H MoS_2_ measured in 730 nm (standard deviation of 2.6%, in A) compared to 455 nm (standard deviation of 7.4% in B). This difference is larger than the expected difference based on the diffraction limit(<2). We can therefore suggest that the flake measured at 455 nm was more spatially heterogeneous than at 730 nm, likely due to subtle differences in the preparation. This can also be visually identified by comparing the flake images, which suggest a ‘clean’ single-domain flake measured at 730 nm, compared to a multi-domain flake at 455 nm. Critically, however, all experiments presented using different above-gap energy light (455, 530, and 600 nm) were done on different flakes (Fig. S1), revealing a trend in the acceleration of the phase transition which persists despite this specific example being chosen for the Fig. 2. We can therefore conclude that, within our preparation method, we are sufficiently consistent to not wash out the observed power and wavelength dependence of the discovered photo-redox patterned pathway.

Turning now to the 1T phase, we find at 730 nm (A) a subtle increase in width from 2.6 to ~4%, counteracting the expected trend, which should see the standard deviation reduce to ~1.6%. This step change is significant and we can therefore conclude that at 730 nm (and also in the dark), the phase transition furnishes undesirable additional heterogeneity. We point out, however, that this may well be substrate dependent: Since actual devices using these flakes are not normally made on glass, which has a relatively weak adhesion, strain effect can most likely be more easily triggered, causing wrinkles and distortions to occur more readily. Future studies on different substrates are motivated to explore this effect in more detail. The 1T phase in 455 nm (B) shows a reduction in width from 7.4% in the 2H phase to 4.8%. Since the 1T phase exhibits ~40% of the 2H intensity (see Fig. 2), we would expect a width reduction to ~3% if the heterogeneity of the flake would not change during the reaction. While we reduce the standard deviation, we do not achieve this boundary, indicating that the reaction is under 455 nm illumination causes additional heterogeneity across the flake. Critically, however, this trend is significantly better than the behaviour observed at 730 nm, especially considering that we started of with a more heterogeneous flake at 455 nm.

During long-term experiments (>1 h), necessary to capture the phase transition dynamics under 730 nm illumination, we observed noticeable changes to the intensity at the glass/**n-BuLi** interface of up to 15% over 12 h. These changes match the observed long-term increase observed in Fig. 2 (B, C) in main text and must therefore be correlated. Given that our light sources are stable, the origin of this behaviour must be related to a either a change in the refractive index of the **n-BuLi** solution, or an increased out-of-focus scattering mechanism.

In addition to this slow intensity increase, long-term imaging at 730 nm reveals several ‘landing events’ of several cluster-like particles. These particles are likely formed in solution reactions involving the highly reactive **n-BuLi**. We therefore believe that the long-term increase in reflection intensity is most likely associated with out-of-focus scattering, which our wide-field microscope will be sensitive to.

**2. Layer dependent PL and Raman signature of 2H-TMDs**


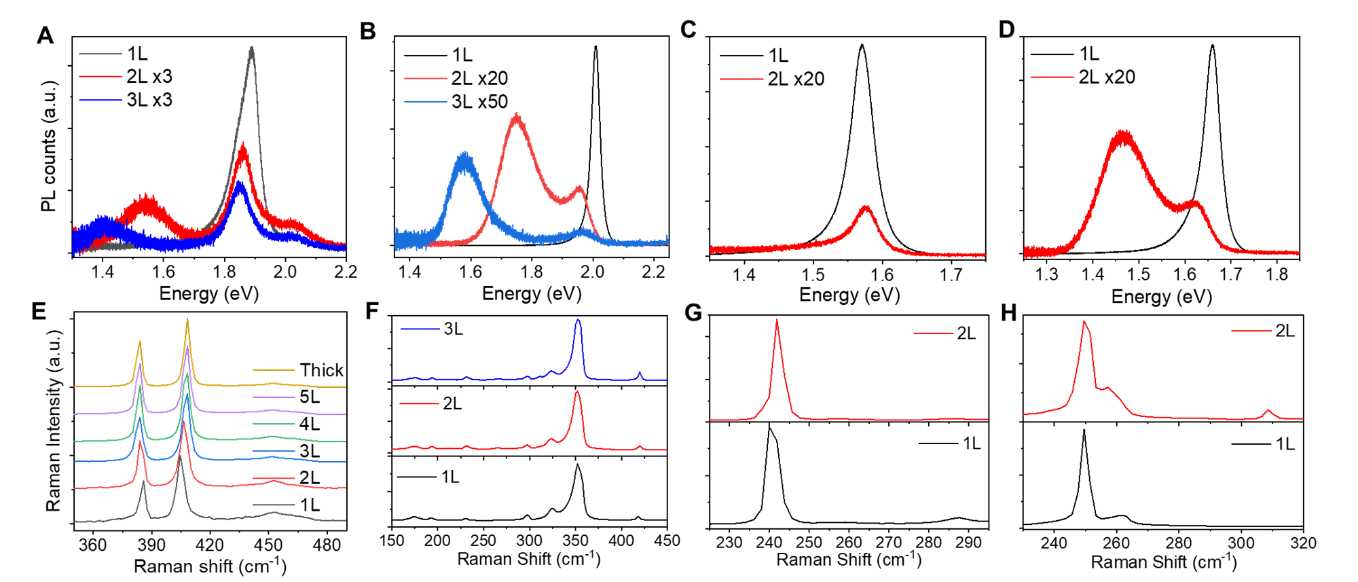


**Fig. S3. PL (A-D) and Raman (E-H) spectra for different TMDs with different number of layer. A,E,** MoS_2_, **B,F,** WS_2_, **C,G,** MoSe_2_, **D,H,** WSe_2_.

Fig. S3 shows the Raman and PL signature measured with different number of layer from each 2H-TMDs, exfoliated on microscopic cover glass for this study. We confirmed the number of layers by comparing the PL spectra reported in previous studies (Table S1). Raman signature from each material with different number of the layers also shows the same trend as reported (Table S2). The position is not exactly the same, but slightly shifted from the different substrates.

| Number of  Layers | Materials | PL signature | ref. |
| --- | --- | --- | --- |
| Mono | MoS_2_ | Single peak around 1.90 eV | ^2^ |
|  | WS_2_ | Single peak around 1.96 eV | ^3–5^ |
|  | WSe_2_ | Single peak around 1.65 eV | ^3,4,6^ |
|  | MoSe_2_ | Single peak around 1.57 eV | ^3^ |
| Bi, few | MoS_2_ | A-exciton emission peak(close to peak from monolayer) with another peak in lower energies, which shifts to lower energy as increasing number of layers | ^2–4,6^ |
|  | WS_2_ |  |  |
|  | WSe_2_ |  |  |
|  | MoSe_2_ | Single peak around 1.54eV (weaker than monolayer) | ^3^ |

**Table S1. PL signature of TMDs with different number of layer.**

| Materials | Raman signature as number of layer increases | ref. |
| --- | --- | --- |
| MoS_2_ | Δ(A_1g_ - E^1^_2g_) increases (blue-shift of A_1g_ mode_,_ red-shift of E^1^_2g_ mode) | ^7,8^ |
| WS_2_ | Blue-shift of the A_1g_(Γ) mode, red-shift of the E^1^_2g_(Γ) mode | ^5^ |
| WSe_2_ | B_2g_ mode (around 309 cm^−1^) active at bilayer | ^3^ |
| MoSe_2_ | Blue-shift of A_1g_ mode_,_ red-shift of E^1^_2g_ mode |  |

**Table S2. Raman signature of TMDs with different number of layer.**

**3. Raman spectra of 1T-MoS_2_**

In Raman spectra, both A_1g_ and E_2g_ peaks are dampened after phase transition to 1T. This has been demonstrated in calculation and experimental works^9–11^. We also observe both peaks are dampened as phase transition to 1T as in Fig. S4, with respect to the n-BuLi treatment duration.


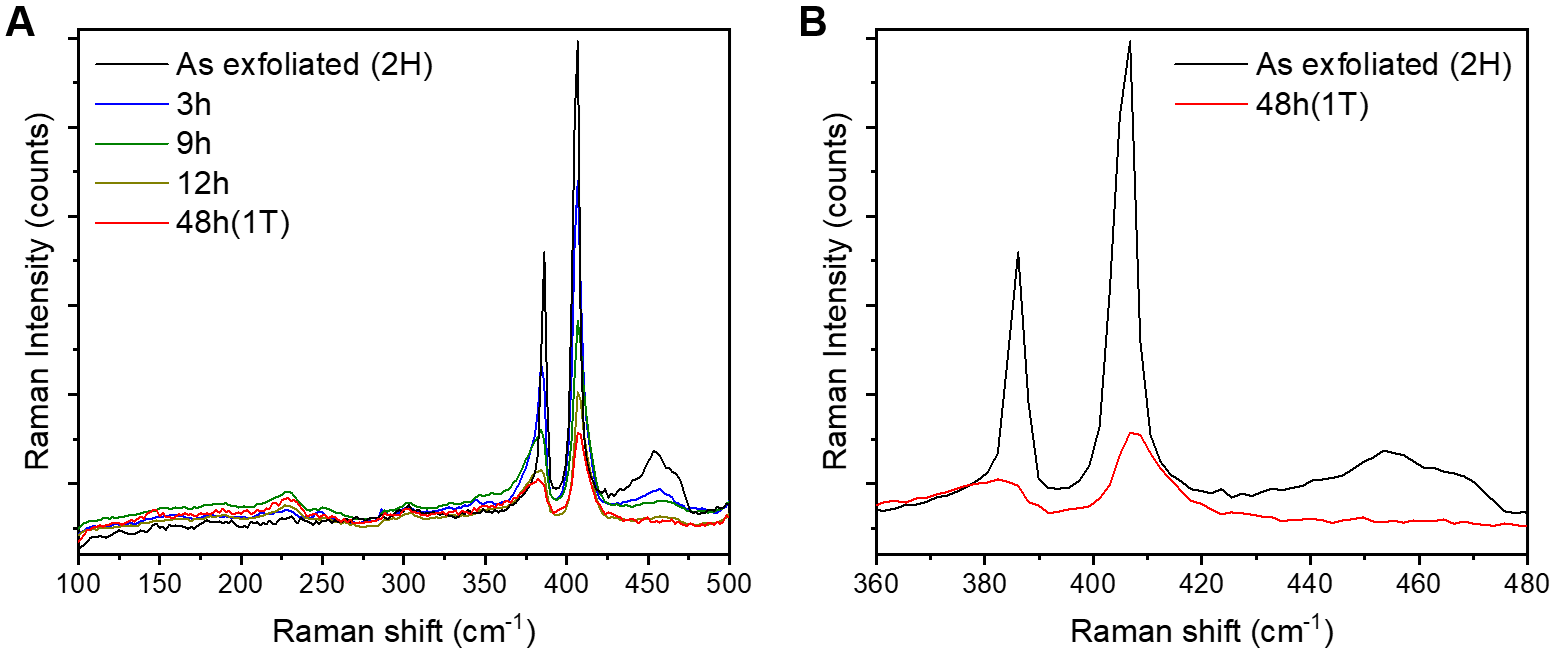


**Fig. S4. Raman spectra of monolayer MoS_2_ with dependent to the n-BuLi treatment time**


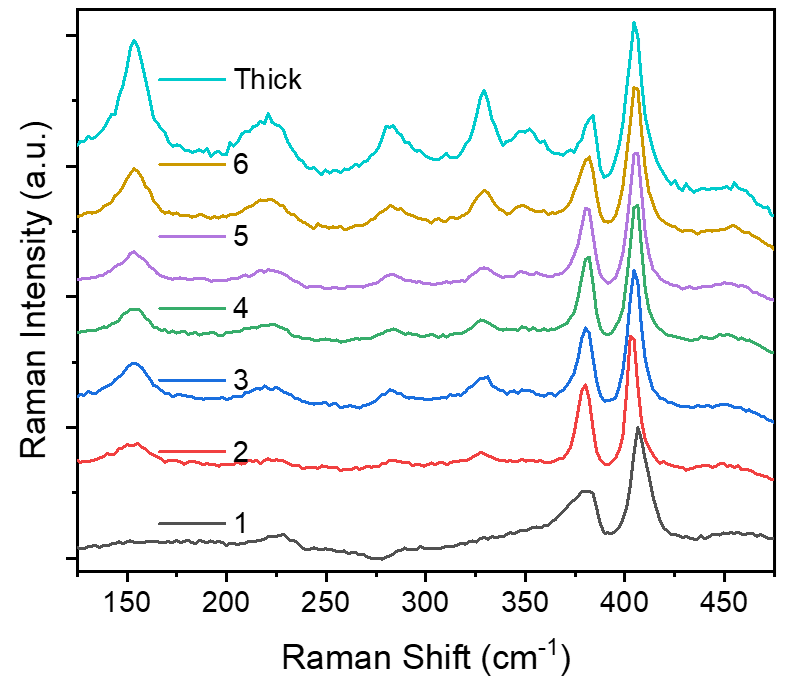


**Fig. S5. Layer dependent Raman signature of 1T-MoS_2_.**

Fig. S5 shows the Raman spectra of 1T/1T’ MoS_2_ with different number of layer. The Raman J-peaks (J_1_≈157 cm^-1^, J_2_≈223 cm^-1^, J_3_≈335 cm^-1^) are getting stronger with increasing the number of layers, while the E^1^_2g_ peak has red-shifted for all layers (E^1^_2g_≈382 cm^-1^)^9,12^.


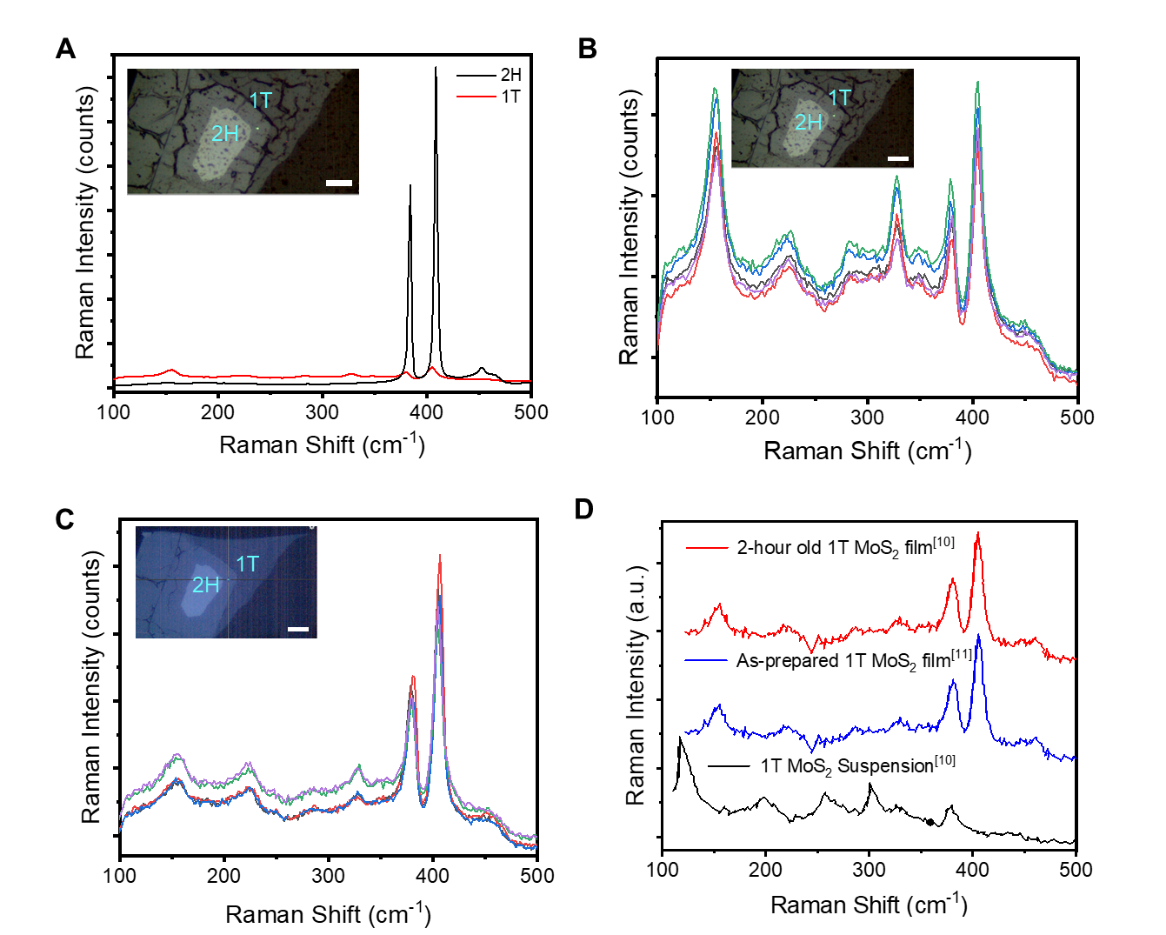


**Fig. S6. Raman spectra with dependent to the washing steps.** **A.** Raman signature in two different region. The outer-ring region changes to 1T phase. (scale bar = 5 μm) **B.** Raman spectra of thick-layered Li_x_MoS_2_ (n-BuLi treatment followed by n-Hexane washing). **C.** Sample in A followed by Raman measurement and further washing using deionized water (DI), Acetone, and IPA. **D.** Raman signature of 1T MoS_2_ suspension and film, data digitized from reference papers^10,11^.

In literatures, the E_2g_ peak is more dampened than the A_1g_ peaks during the phase transition to 1T^9–11,13^.This has been attributed to the metastable nature of 1T MoS_2_ in air^14^. Furthermore, 1T MoS_2_ synthesized from the bulk powder in aqueous suspension exhibits dramatically damped E_2g_ and A_1g_ peaks^10,11^, which rapidly arise again at freshly prepared film with water and air exposure^10^ (Fig. S6 D). We examined this washing effect in bulk MoS_2_. In Fig. S6 A-C, we treated a very thick flake with n-BuLi for 10 h and washed the sample mildly with n-Hexane before we measured steady-state Raman spectra. Here, we observe clear wave front of two phases similar to our observation in the main text and in literature^15^ (optical image in Fig. S6 A). The Raman spectrum of 1T phase (outer ring) also shows damped and red-shifted signals of E_2g_, and A_1g_ peak compared to an as-exfoliated sample in the 2H phase (Fig. S6 A). The Raman signal of lithiated MoS_2_ in 1T phase (Fig. S6 B) shows lower E_2g_, A_1g_ peak intensities with higher J-peak intensities (even higher J-peaks ratio than fresh-restacked film in D^11^). However, as we further washed the sample with deionized water (DI), Acetone, and isopropanol (IPA), in Fig. S6 C, both E_2g_, A_1g_ peak gets higher while the J-peak intensity decreases. The Raman spectra from fully-washed sample are similar to 2-h old restacked film in the literature (red curve in Fig S6 D) ^11^.


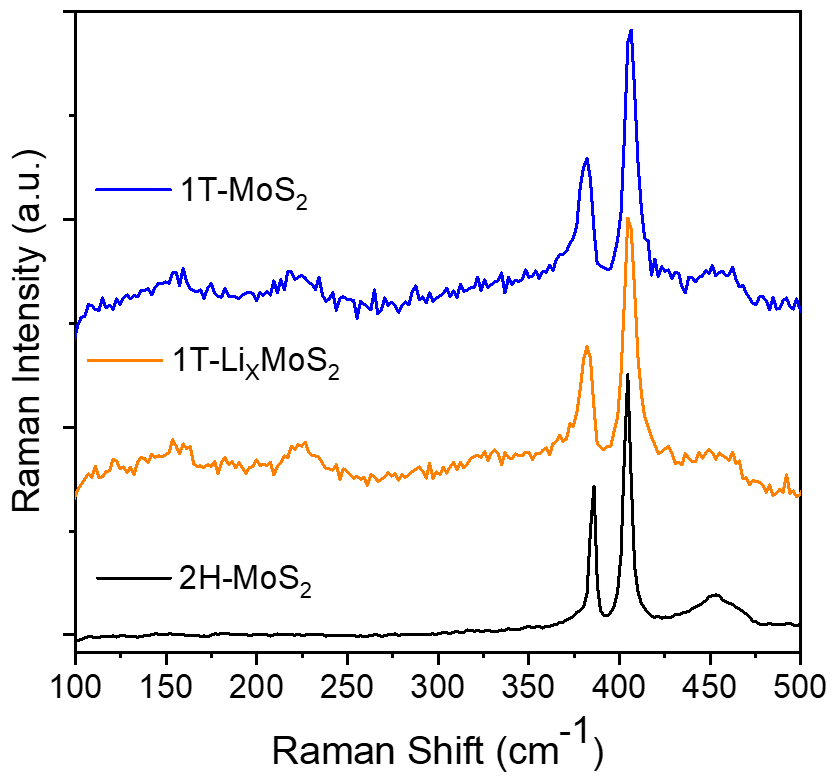


**Fig. S7. Raman graph of bilayer 1T-MoS_2_ with different washing step.** (Hexane washed sample refers to 1T/1T’-Li_X_MoS_2_, and, DI/Acetone/IPA washed sample refers to 1T-MoS_2_).

Fig. S7 shows the Raman signature of bilayer 1T/1T’-MoS_2_ with different washing steps using Hexane (1T-Li_x_MoS_2_) and further washing with DI/Acetone/IPA (1T-MoS_2_). After phase transition in bi-layer MoS_2_, we firstly washed the sample using n-Hexane, which remains lithium cation within the 1T structure(1T/1T’-Li_X_MoS_2_)^16,17^. Then, second step of washing followed using DI, Acetone and IPA for removing lithium(1T/1T’-MoS_2_). The two graphs have very similar spectra having same red-shifted of E^1^_2g_ and A_1g_ peak and emergence of J-peaks. Therefore, we concluded removing the lithium has negligible effect on Raman signal in bilayer 1T-MoS_2_.


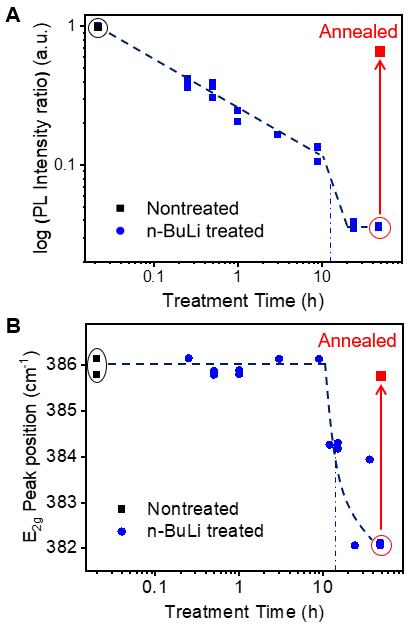


**Fig. S8. Ex-situ Raman analysis for monolayer MoS_2_ treated with n-BuLi with different time. A.** A-exciton PL (around 660 nm) intensity. **B.** Raman E_2g_ peak position. All sample has been washed using deionized water, Acetone, and IPA.

For tracking the dynamics of *ex-situ* Raman signal, we monitored E_2g_ mode comparing with PL spectra, using exfoliated monolayer in Fig. S8. Fig. S8 A shows the ex-situ dynamics of A-exciton PL intensity and B shows the shift of E_2g_ peak position with n-BuLi treatment duration. The PL dramatically quenched during the semiconductor (2H) to metal (1T/1T’) transition at first 12 hours. Especially, when PL drops significantly, we can observe the red-shift of E_2g_ peak (Fig. S8 B). We note that, the red-shift of 3.8 cm^-1^ is as same as we achieve by photo-redox patterning (3.9 cm^-1^), showing the robust nature of this feature in assigning the phase transition. This shift is also similar to the literature which showed red-shift of E_2g_ peak about 2.7 cm^-1^ at 2H to 1T phase transition^18^. Therefore, we used the shift of E_2g_ peak as a second major indicator for Raman analysis together with the existence of J peaks throughout the study.

The quenched PL and red-shifted E_2g_ peak reversed back close to the original intensity and position when thermal annealing (300℃, Ar/H_2_ atmosphere). This shows reversibility of the phase transition.

**Analysis on J-peaks**

The emergence of J-peaks is a well-established identification of 1T/1T’ phase of TMDs as it has been confirmed after n-BuLi treatment (as in our study)^19–22^, during electrochemically lithiation^23,24^ and for chemically grown samples^25^. However, J-peak shows substantial variation in both position and intensity depending on the sample conditions, which include the starting material form (crystal, powder, chemical vapour deposited), lithiation method, effect of washing steps, air exposure and measurement parameters. The Raman spectra of 1T-MoS_2_ exhibits variation in J-peaks in crystal^22^, drop-casted film^26,27^, CVD (chemical vapor deposition)-grown film^19,20^, nanostructures^28,29^, when synthesized form bulk powder^30,31^, and mechanically exfoliated sample^32^ Especially, mechanically exfoliated, or CVD-grown mono, and few-layer MoS_2_ samples show a very similar Raman signature compared to our data in Fig. 1D. Moreover, we can observe both E_2g_, A_1g_ peaks having comparable or higher intensity than J-peaks similar to our data from thicker layer (Fig. S4 B).


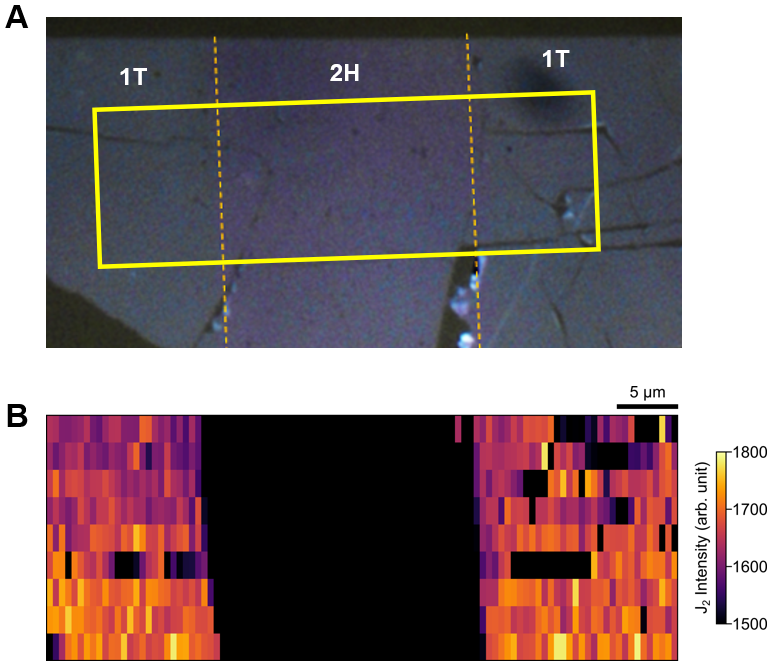


**Fig. S9. Raman mapping for photo-redox patterned monolayer MoS_2_. A,** Optical image of patterned flake. **B,** J_2_ peak intensity map from the yellow-square region in **A**. Sample has been washed using deionized water, Acetone, and IPA.

In Fig. S9, we show the optical and Raman J_2_ peak intensity mapping of the photo-redox patterned flake. Notably, the optical microscope image in Fig. S9 A shows optical contrast of 1T and 2H region is quite different. As the flake becomes metallic, the phase-patterned 1T shows a grey colour while the 2H flake appears somewhat red.

As the J_2_ peak is the most intense peak in our 1T monolayer, similar to what was reported for exfoliated samples^32^, we monitored J_2_ peak as our representative indication for J peaks. Fig. S9 B shows the peak intensity and position of J_2_ peak. As discussed earlier, the peak intensity shows pronounced variations of ± 20% (95% confidence interval). even within the same chemical treatment and environment. The direct quantitative comparison of J peak intensities is not a very reliable metric as it originated from the 2 x 1 superlattice structure of distorted 1T phase and the position and intensities shift substantially depending on environmental factors, as shown in different literature examples^9,11,14^. Despite this complication, Raman spectroscopy can still be used to identify the metallic phase of MoS_2_.

We conclude that the dampened Raman E_2g_, A_1g_ mode with emergence of J-peak can be indicator of 1T phase while it has some variation in its intensity and position, and the shift of E_2g_ peak could be used to monitor the phase transition from 2H to 1T.

Taken together, as there are J-peaks (while even slightly in different intensity) and the identical red-shift of E_2g_ peak in both for dark (48h n-BuLi treated sample without light) and photo-redox patterned sample, we conclude that both sample undergoes the same phase transition process to 1T.

It is worth noting that we have monitored our real-time phase transition inside an air-tight cell, but measured Raman in ambient conditions. Furthermore, for safe ex-situ measurements with stable signals, samples were washed out multiple times using Acetone, IPA, and DI, for removing the residue of n-BuLi, organic and lithium and lithium oxides on the surface. Without proper washing, the thin layer of MoS_2_ gets easily covered with think organics and lithium oxide layer by air exposure. This process would effects the sample to have stronger E_2g_, A_1g_ peaks and lower J peaks^10,11^.

**4. Optical absorption properties of MoS_2_**


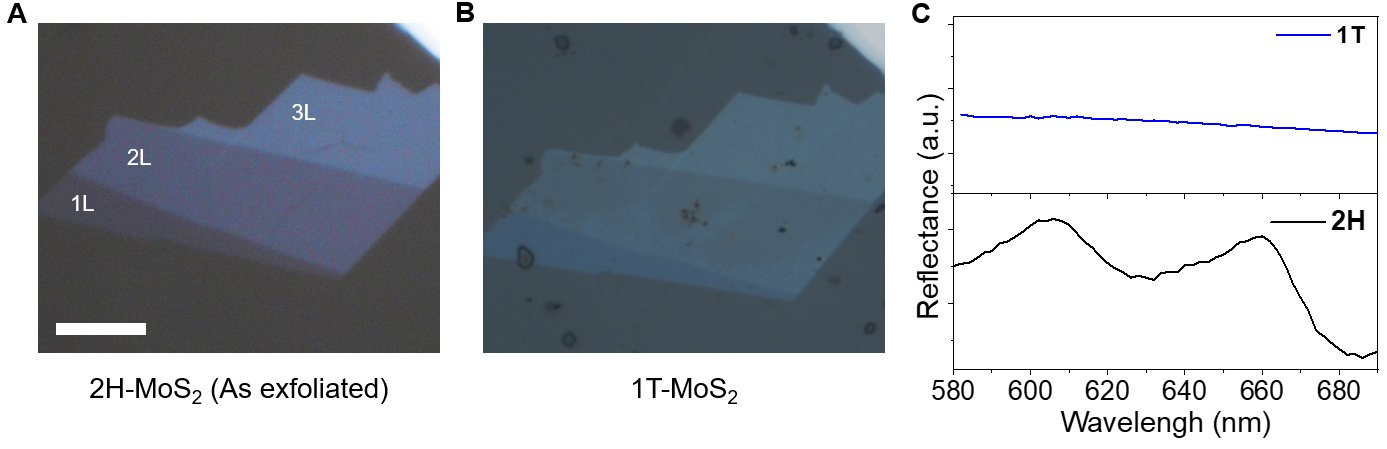


**Fig. S10. A,** Optical image of 2H-MoS_2_ (scale bar = 10 μm), and **B,** 1T-MoS_2_ **C,** Reflectance spectra of the 1T and 2H phase MoS_2_.

Fig. S10 A, B shows the steady-state optical image of 2H and 1T-MoS_2_ taken with optical microscope using a 100× air-objective. It shows the optical contrast has decreased as it changed to metallic phase. Fig.S15 C is the reflectance spectra of 2H and 1T/1T’ phase, the two excitonic peak from semiconducting properties (~602 nm, ~660 nm) has disappeared in 1T/1T’ phase^15,33^.


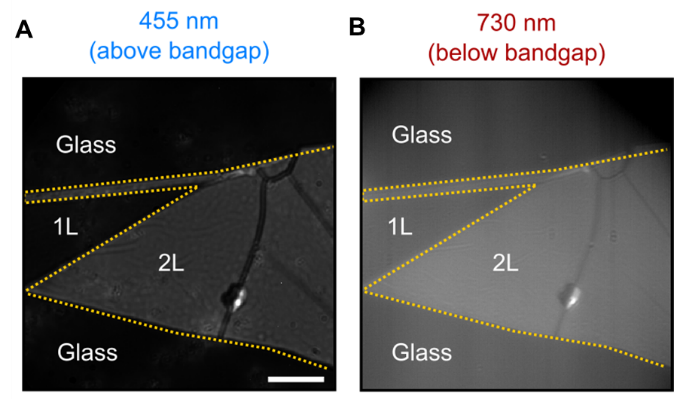


**Fig. S11. Optical image of WS_2_ with different illumination energy.** **A,** 455 nm (above the bandgap). **B,** 730 nm (below the bandgap) illumination (scale bar = 5 μm).

Fig. S11 shows the optical image using two different illumination wavelengths of 455 nm±10 (Blue) and 730±10 nm (Red). The image taken at 455 nm has a higher contrast than the image taken with 730 nm due to an increased optical absorbance at this wavelength. The optical absorbance peak of WS_2_ locates at around 516 nm (B-exciton, 2.40 eV), 613 nm (A-exciton 2.02 eV), and 440 nm (C-exciton, 2.83 eV)^34^. This optical absorption also could induce optically generates electron which induced to the chemical reaction process to TMDs^35^.

The illumination power in this study is less than 1 W cm^-2^ for photo-redox phase enginering. The power density used in our work is also significantly lower than the damage threshold, which is exploited in layer-by-layer etching, or trimming of the MoS_2_. This occurs at power densities of approximately 3981^36^, 625000^37^, 21000^38^ for MoS_2_, and 26875 for MoTe_2_^39^ (unit : W cm^-2^).

Moreover, a power density of 1 W cm^-2^ is low enough to not significantly affect the intrinsic material properties of TMD, as highlighted in literature, where the PL of monolayer MoS_2_ remained unchanged for up to 50 min when continuously irradiated at 532 nm laser with a power density of ~1769.3 W cm^-2^, i.e. 1000-fold higher then this study. According to the same study, bilayer MoS_2_ is not affected until a power density of ~2653.9 W cm^-2^ for up to 305 min^36^. Therefore, we can conclude that laser density of 1 W cm^-2^ has negligible effect on the material. Furthermore, from our new wavelength dependent measurements, we find that 532 nm exhibits the same mechanism as 455 nm, i.e. represents C-exciton excitation (section 5).

Based on the fact that our photo-detector exhibits Ohmic-like contact properties produced in under 10 seconds for monolayer MoS_2_, the photo-redox phase engineering strategy at least up to a power density of 1 W cm^-2^ is applicable. Moreover, when we change the chemical to PAH-Li system, the reaction becomes even faster approach to achieve phase transformation, by combining illumination with a polyaromatic organo-lithium system (section 16).

Therefore, as we are at very low-power range less than 1 W cm^-2^, we are confident that our method as does not modify the intrinsic properties of the flake and chemical system.

**5. Wavelength dependent phase transition of MoS_2_**

In extended figure 1, we provide data measured with 600 nm and 530 nm for supporting wavelength and power dependence on phase transition reaction with data in Fig. 2 (measured in 455 nm, 730 nm, and dark). We have chosen different wavelengths following the optical absorption spectra for MoS_2_. The absorption spectrum of monolayer MoS_2_ shows clear signature of excitonic resonances at ∼650 nm (A-exciton), and ∼610 nm (B-exciton), and ∼450 nm (C-exciton)^40^. The C exciton originates from the band-nesting effect, which is independent to the excitonic resonance^35^, and absorbs more strongly than the B and A excitons. Between the B and C exciton, the absorbances reduces (between ∼500-590 nm).

We firstly chose 600 nm to explore potential differences between the C and B excitonic resonance. We note that the A and B excitons are qualitatively similar in nature, and we therefore focused only on the high-energy B-exciton in this study. Secondly, we explore a nominally off-resonant illumination at 530 nm to verify the effect of optical absorbance on this process for correlating the optical absorbance to phase transition speed.

**5.1. RICM measurements at 600 nm.**

600 nm excites the B-exciton, which shows a high exciton binding energy and has a direct bandgap for the monolayer in 2H-MoS_2_. We find that optical excitation of this exciton during phase transition in n-BuLi leads to an accelerated phase transition compared to below-band gap illumination and dark conditions. However, the acceleration is significantly lower than for 455 nm illumination, which excites the C-exciton of MoS_2_. This trend applies for both mono and bilayers. Notably, throughout the experiment, the phase front remained sharp indicating charge transfer limitations


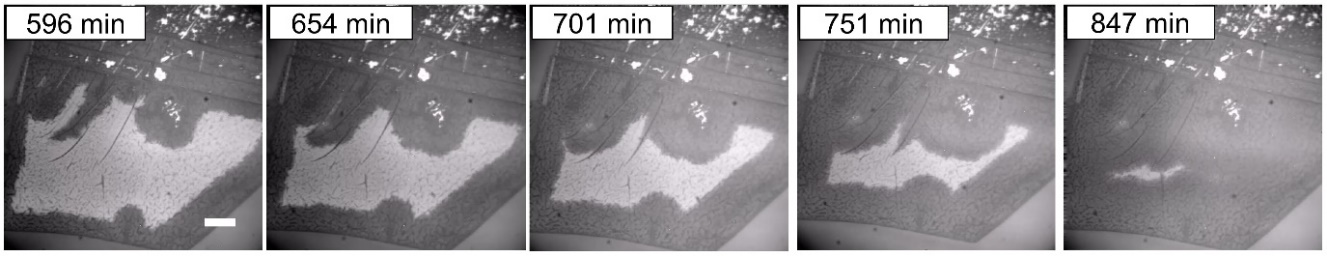


**Fig. S12. Snapshot of the phase transition in bilayer-MoS_2_ visualized at 600 nm.
(Scale bar = 5 μm)**

Firstly, we measured a large bilayer with no monolayer surroundings. The flake was first kept in the dark for 596 min, to evaluate the progression of the phase transition under dark conditions. Afterwards, we illuminated the flake with whitelight filtered by a 600±40 nm bandpass filter and a power density of 360 mW cm^-2^. This power density was chosen to excite the same number of charge carriers as at 455 nm, 110 mW cm^-2^ for a direct comparison.

As shown in Fig. S12, we observed a clear shrinking core type behavior with a sharp wavefront at dark. This behavior closely resembles the phase transition mechanism observed at 730 nm, suggesting it is charge-limited (730 nm, see Fig. 2A-B). Here, the edges and cracks across the flake act as ‘active’ lithiation sides and allow the phase transition to proceed.

Then, we measured *in-situ* and the velocity of sharp wave front was measured by ∼0.24 nm s^-1^. Comparing to the wave front speed of below-gap illumination (730 nm), which is ∼0.08 nm s^-1^, it is 3 times faster. Illumination at 600 nm thus accelerates the intrinsic reaction mechanism what we measured in 730 nm (charge-limited). We also note the absence of any wrinkles.


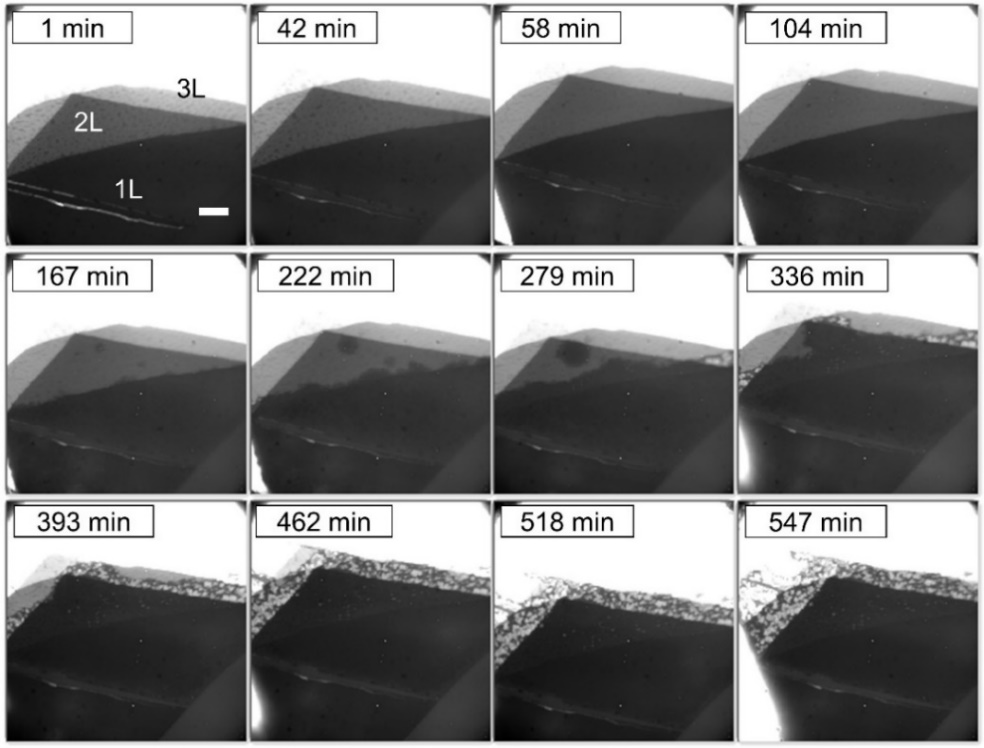


**Fig. S13. Snapshot of the phase transition in terraced-structure MoS_2_ visualized at 600 nm.** (Scale bar = 5 μm)

For studying the structure and layer dependence, we conducted second measurement using 600 nm illumination with a terraced-structured MoS_2_. Fig. S13 shows the phase transformation of this terraced structure, which shows similar 2L wavefront speed of ∼0.32 nm s^-1^.

**5.2. RICM measurements using 530 nm.**

The wavelength of 530 nm is above the bandgap, but the optical absorbance is lower than 600 nm. Therefore, we monitor the reaction using slightly higher power than 600 nm (510 mW cm^-2^) to compare the reaction in similar optical absorbance.


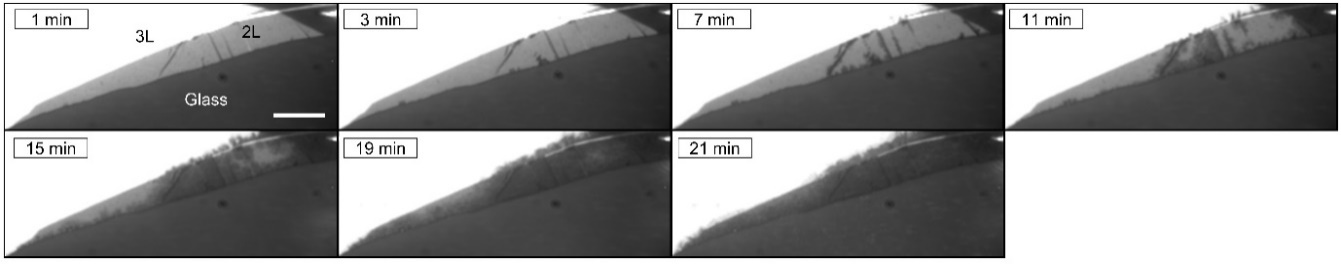


**Fig. S14. Snapshot of the phase transition in MoS_2_ visualized at 530 nm.** (Scale bar = 5 μm)

At 530 nm, the phase transformation was accelerated compared to 600 nm and proceeded with more diffuse phase fronts, similar to 455 nm low power illumination, suggesting a deviation from a strict charge-transfer process (Fig. S14). 530 nm excitation thus behaves like a C-exciton excitation, albeit still slower.


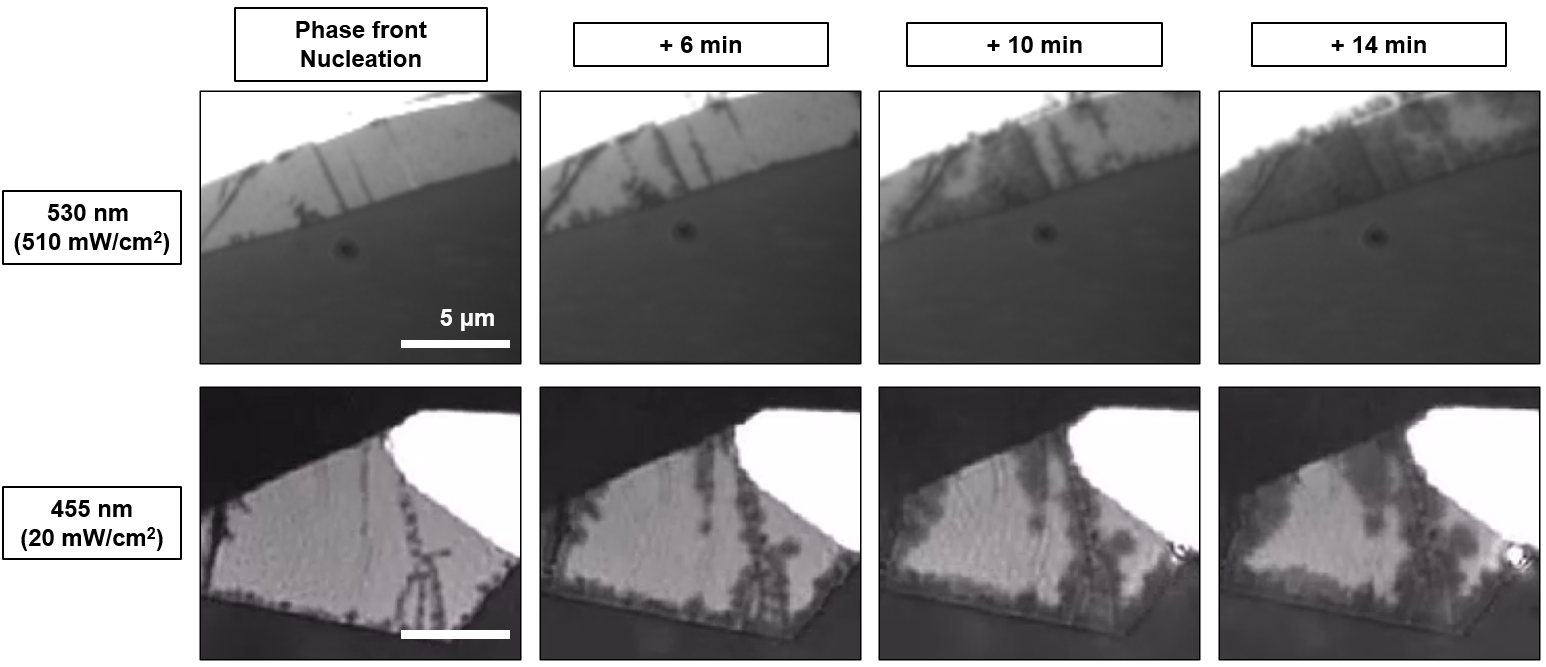


**Fig. S15. Comparison of the phase transition in 2L MoS_2_ visualized at 530 nm (top) and 455 nm (bottom).**

In Fig S15, we compare experiments using 530 nm (510 mW cm^-2^) with 455 nm (low power, 20 mW cm^-2^). Clear similarities are revealed both in terms of phase front speed and in terms of the sharpness of the phase boundaries. 530 nm therefore behaves like C-exciton excitation, but slower. This is most likely due to the 0.38 eV lower excitation energy, imposing an excited state barrier on the generation of free charges. More theoretical studies of the exact electronic structure of the C-exciton are required to fully reveal the nature of this effect.

**5.3. Wavelength-dependent photo-redox phase transition speed.**

Taken together these results (Fig. S12-15) demonstrate that the critical part toward accelerating the phase transition is the availability of the material to create free charges under photo-excitation. Here, the C-exciton outperforms the B-exciton (also the A-exciton given their close electronic nature), which we attribute to band nesting effects.

The phase front speeds retrieved from these studies with absorption of MoS_2_ are reported on **the extended data 1** showing that above-bandgap excitation always improves the reaction speed, but that the underlying excitonic structure plays an important role in the absolute acceleration that can be achieved.

The optical absorption in **extended data 1** (black curve) have measured by micro-ellipsometer. The ellipsometry angles were fitted to a thin film model to extract the dielectric constant $\varepsilon$of the monolayer MoS_2_. The absorption of the monolayer was then calculated following the equation: $Re[-1i\frac{2\pi}{\lambda}d\varepsilon]$. The absorption spectrum of monolayer MoS_2_ shows clear signature of excitonic resonances at ∼650 nm (A-exciton), and ∼610 nm (B-exciton), and ∼450 nm (C-exciton)^40^. Decomposing the absorption spectrum into individual exciton resonances guided us to select wavelengths to study.

The optical illumination using 600 nm (360 mW cm^-2^), 530 nm (510 mW cm^-2^), and 455 nm (20 mW cm^-2^) shows similar wavefront speed as 0.3 ~ 1.3 nm s^-1^, compare to dark (0.08 nm s^-1^) and 455 nm in high power (510 mW cm^-2^, 110 nm s^-1^). However the mechanism of above gap illuminations (530 nm, 455 nm ) and excitonic resonance (600 nm) and below gap illumination (730 nm ) is different. B-exciton excitation behaves qualitatively similar to 730 nm, with a charge transfer limited reaction mechanism.

This behaviour can be attributed to the large exciton binding energy (>0.5 eV), effectively preventing free charge generation. Nonetheless, a mild light effect is revealed suggesting that photoexcitation leads to a small fraction of free charges that can partake in the phase transition. Conversely at 455 nm and 530 nm, the C-exciton resonance dominates the behaviour. In comparison, this strongly supports a higher charge carrier generation yield for the C-exciton compared to the B-exciton, as postulated in previous studies. Exciting at lower C-exciton energies slows the photo-redox phase patterning reaction down, but not in proportion to the overall absorption cross section, suggesting an excited state barrier to charge generation.

**6. Image analysis for structural dependent phase transition**

**6.1. Different nucleation point across the flake – Edge dependence**

Fig. 2C reveals a pronounced difference in the phase conversion onset (or phase nucleation) dependent on whether the intercalation originates from a glass/bilayer edge, or a monolayer/bilayer terrace. Furthermore, Fig. S16 shows the discrepancy of phase transition in different section of the flake. All flakes show the discrepancy of phase transition in different section of the flake. The middle region in the Figure in below (dashed-blue line) shows faster reaction then other regions (marked with star). Given that all three flake segments have been prepared similarly and exhibit the same glass/interfaces, this discrepancy must arise from different activation barriers along these interfaces (reasons may include defect density, strain, substrate interaction variations, etc.). Importantly, while the time for phase nucleation differs, once nucleated, the phase front velocity is the same for all flakes, as also shown in Fig. 2C in the manuscript (identical slope).


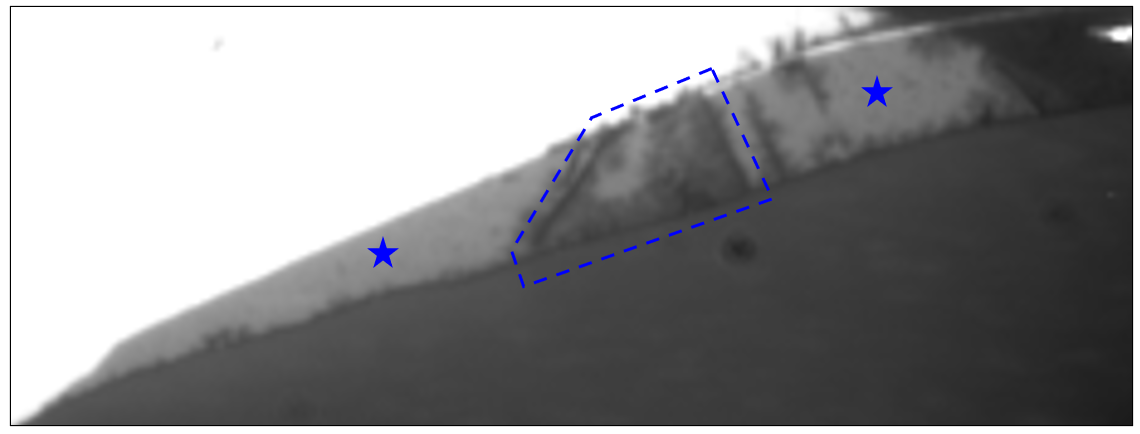


**Fig. S16. Snapshot of the phase transition in MoS_2_ visualized at 530 nm after 11 min.**

Literature calculation studies further propose that the phase transition reaction is more favorable to specific edges, such as the bare Mo-edge or the S-edge with 50% S^41^. Therefore, we can postulate that different types of edges can cause different activation barrier that determines the time for nucleation of the new phase.

The images using above-gap illumination (Fig. 2D) provides a comparable scenario whereby the top left part of the flake is an edge (glass/2L), while the bottom right a Terrace (1L/2L). From the images in Fig 2D, we find that the triangular edges of the bilayer flake and strong defect sites in the center act as primary nucleation sites, as shown in red circle in Fig. S17.


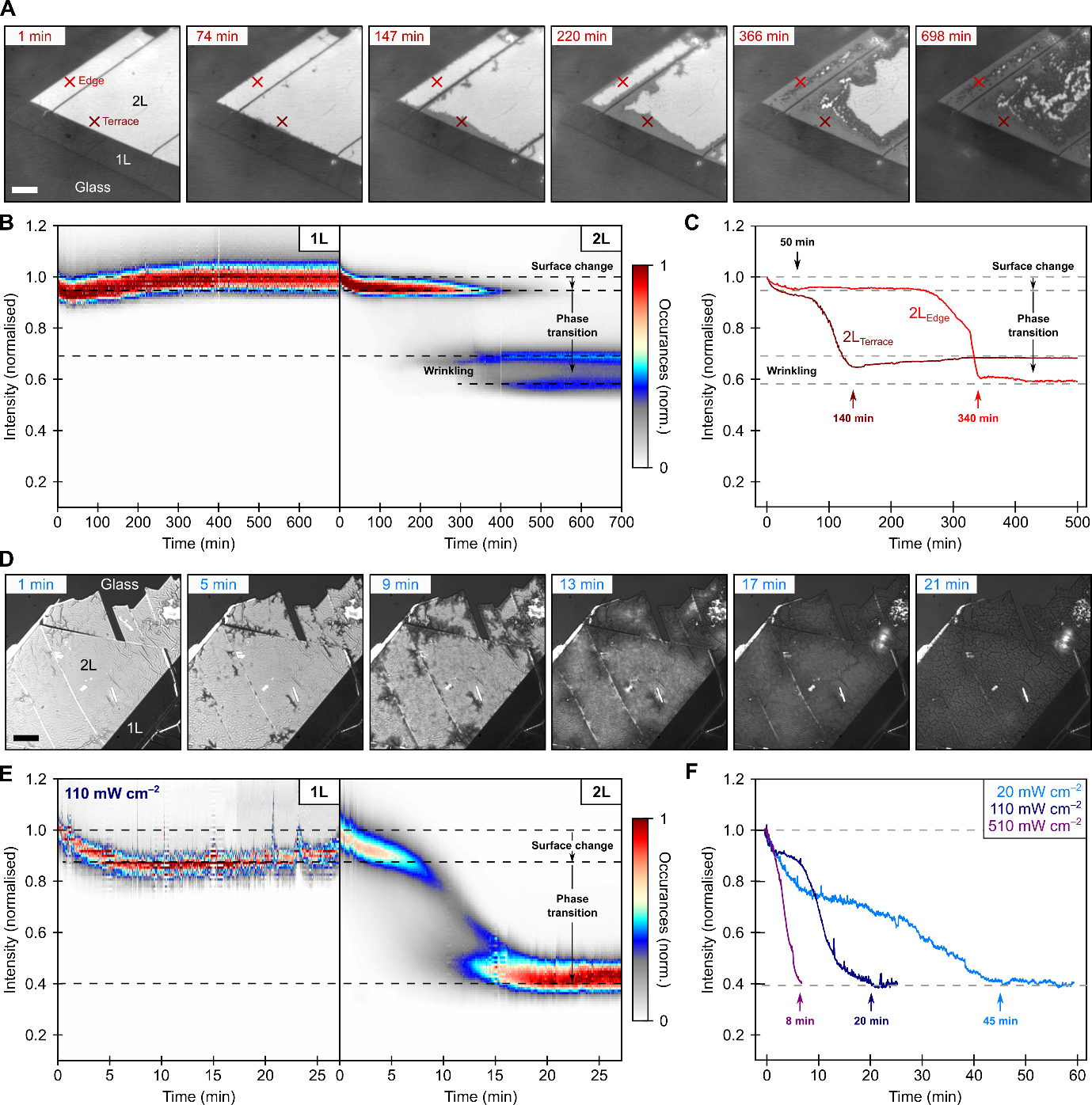


**Fig. S17. Visualisation of several 1T nucleation sites (red circles) in 2L under 455 nm illumination** (adapted from Fig. 2D in manuscript).

Notably, all nucleation sites indicated above occur roughly at the same point in time with no significant delay irrespective of whether the structure is a terrace or an edge. Further, once nucleated, the phase transformation proceeds through the phase, pushing a (diffuse) phase boundary along until the entire flake is converted. This behaviour is markedly different than the flake examined under 730 nm illumination, where the 1L terrace nucleated first along the terrace, rather than in its corner. These results suggest that photoexcitation provides sufficient energy to overcome the difference in nucleation activation energy barriers between edges and terraces and that it prefers nucleation along exposed (‘pointy’) features of the flake.

**6.2. Thickness dependence**

To explore how the observed photo-redox process occurs for thicker samples of more than 2L, we conducted experiment on thicker flakes:

- At 600 nm, we explored a terraced structure with increasingly thicker layers (Fig. S13).

We observed nucleation of the 2L with a single wavefront moving from the bottom side of the image across the whole layer, independent to the numbers of layers. This suggests a layer-by-layer approach between successively thicker layers. Importantly, this highlights that even thick layer structures can be converted more rapidly with light exposure in such structure, as the mechanism remains the same, that is, the activation barrier toward nucleation is lowered across the surfaces, helping to move the phase front along.

Two caveats remain: Firstly for the thicker layers in this experiment we observe more wrinkles and in some other cases even delamination, most likely due to internal strains imposed by the surrounding environment. Secondly a bulk sample with no terraces will not benefit from the photo-redox effect as much as a terraced structure.

- At 530 nm, we carried out measurements on a terraced structure with a large 3-4L flake as shown Fig. S18.

As before, the 1T phase nucleates at corner of the 3-4L flake (right hand side in the Fig. S18) and move through this nucleation point across the flake. Interestingly, after about 84 min, we observe distinct intensity regions deep inside the flake, labelled ‘a’ (orange) and ‘b’ (purple), which connect the 2H and converted 1T phase. These intermediate region likely correspond layered structures in which 1 or 2 of the 4L structure exhibit 1T character. We highlight that for a large 2L structure illuminated at 600 nm no such phase terracing was observed.


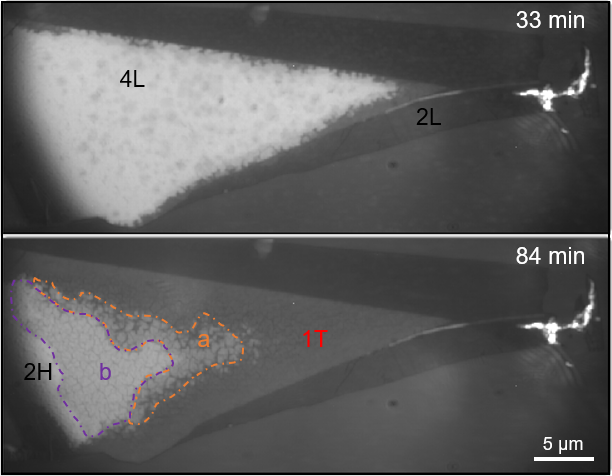


**Fig. S18.** **Snapshot of the phase transition in terraced-structure MoS_2_ visualized at 530 nm.** (Scale bar = 5 μm)

These results outline that the photo-redox phase transition still applies in thicker flakes but proceeds via a substantially more complex mechanism that depends on the terracing of the structure. This effect may be used to vertically inscribe heterogenous 1T/2H structures, but more research needs to be conducted to explore the optimised parameters for this process. Critically, it highlights that the effect of illumination is not just confined to the surface, but proceeds also via a bulk effect.

**Generalisation to thicker systems:** To enable rapid conversion of thick flakes in simpler way, photo-redox pathways alone are not sufficient. Instead, we show in our manuscript how redox matching can be utilised to ensure such samples can be converted.

**7. Initial dynamics of phase transition in bi-layer MoS_2_ from steady-state PL and Raman spectra**


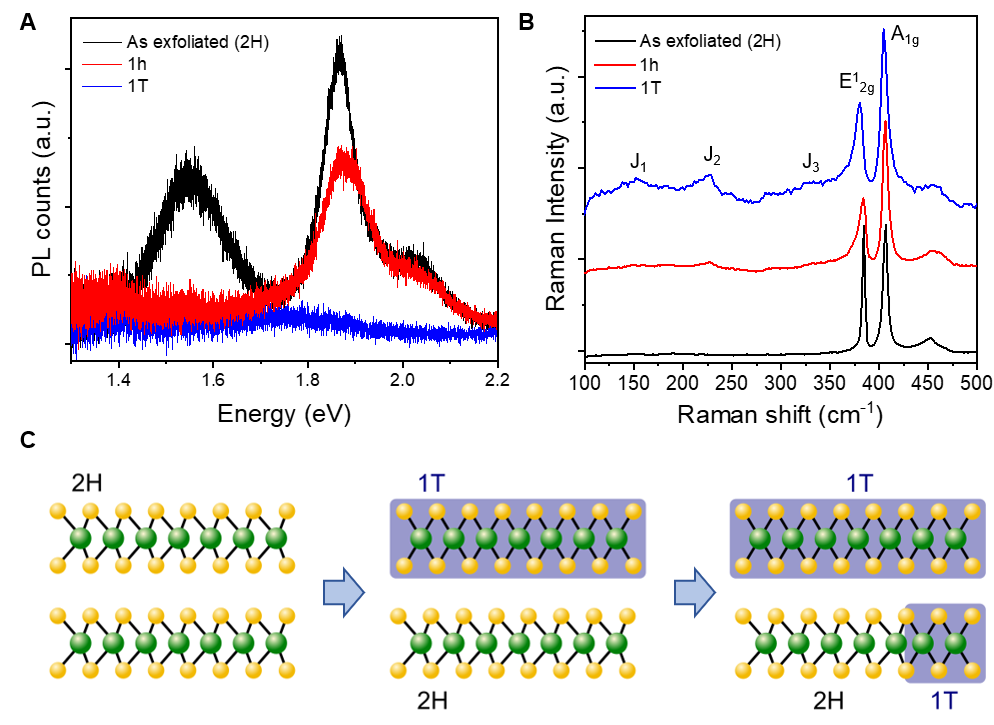


**Fig. S19** **Phase transition mechanism of bi-layer MoS_2_ by steady-state PL and Raman analysis.** **A,** PL spectra of 2H, 1T and sample treated with **n-BuLi** for 1 h **B,** Raman spectra of 2H, 1T, and 1 h **n-BuLi** treated sample **C,** Schematics of initial dynamics for phase transition.

Fig. S19 A, B shows the steady-state measurement of initial dynamics happening in 1 h without illumination (dark). In Fig. 2B the darkening of the bilayer flake stays until around 400 min and the wavefront starts to show from 300 min. Also, initial rapid darkening happens within 50 min. Therefore, we carefully treat **n-BuLi** on bilayer MoS_2_ for 1 h to examine the structure change at initial stage.

The PL shows monolayer-like single peak having its maximum around at 1.84 eV (red graph in A). This indicated dramatic weakening of van der Waals interactions between the layers and each layer reacts as independent single layer. Previous reports show strain induces direct to indirect bandgap transition to mono-layer^42^, or a red-shift of the two PL peak to the bilayer MoS_2_^43^. However, this PL spectra(red graph in A) shows a single peak position of 1.84 eV which corresponds to the recombination of the negative trion^44^. Although the PL intensity is very low compare to intrinsic monolayer because the dominant trion recombination pathway is nonradiative^45^, this single PL peak suggests the van der Waals interaction has weakened. In Raman spectra, the 1 h sample shows the shift and broadening of E^1^_2g_ peak and A_1g_ peaks, due to the charging of the structure^12^. However, the J-peaks, which is relating to the distortion of the structure by lithium cation is not showing yet^9^.

In electrochemical lithiation, it has been reported the opening of the layer structure for lithium intercalation starts at 1.5 V vs. Li/Li^+46^. This also could be matched with the initial darkening and monolayer-like single PL, correlated with initial expansion of interlayer spacing before the lithium cation gliding through the gap. This steady-state optical characterization result of the first darkening of the flake in Fig. 2B is highly corresponding to the weakening of the van der Waals interactions and prior phase transition of top-most layer, which is also reported as thermodynamically favourable from the calculation^47^.

**8. Photo-redox diffraction limited phase patterning of MoS_2_**

**
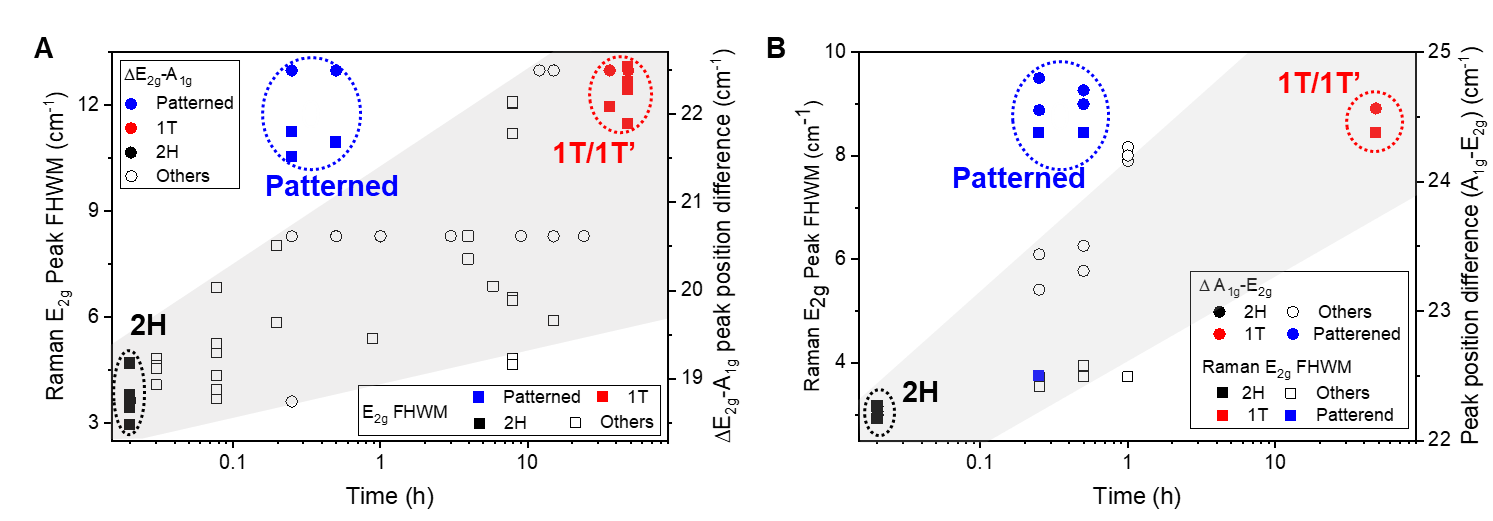
**

**Fig. S20.** **Raman analysis of phase transition of MoS_2_ with photo-redox phase-patterned flake. A,** monolayer, and **B,** bilayer.

Fig. S20 shows the statistics of Raman graph. For both monolayer and bilayer, the full width at half maximum (FHWM) of E_2g_ Raman peak increases and peak position difference between E_2g_ and A_1g_ Raman peak gets wider as the 2H phase converts to 1T by red-shift of E_2g_ mode. . Also, we show J_2_ peak intensity mapping data for photo-redox patterned monolayer in Fig. S9 (See section 3 in SI). The Raman analysis using different exfoliated samples shows this phase transition reaction in dark takes more than 24 h for both mono- and bi- layer (red circled region for A, B). Our phase-patterned flakes shows the same Raman changes happens less than 20 min, which shows the effective photo-redox phase engineering (blue circled region for A, B).

The extended figure 2 shows the microscopic PL image from the flake in Fig 3A, using a 532 nm excitation and imaged on the EMCCD camera with 660±10 nm bandpass filters for measuring A-exciton PL. The strong uniform PL form non-illuminated monolayer shows the semiconducting properties of non-illuminated region. Currently, phase patterning in TMDs is either achieved by direct laser writing or an electron beam lithography^19,21,33,48^. The former method requires specialized femtosecond lasers or extremely high laser powers, while the latter suffers from the need for a precisely controlled electron beam and stable masking reagents as well as using the n-BuLi for several days^19,21^.

**9. Photoresponse of MoS_2_ photodetector**

All the photodetectors are working at photoconductor mode with V_DS_ = 1 V. Fig. S21-22 illustrate the detailed power and wavelength dependence of 2H-MoS_2_ and phase engineered-MoS_2_ photodetectors respectively. Both devices shows strong power- and wavelength-dependent photocurrent values which is in line with literature reports^49–52^. With similar scales of power density, we observe that the phase engineered-MoS_2_ shows higher photocurrent than the non-treated 2H-MoS_2_. The photocurrent also shows reproducibility with illumination on/off cycles. We calculated the responsivity (***R***) using following equation:

$$\boldsymbol{R}=\frac{I_{ph}}{PS}$$

Where $I_{ph}$ is the photocurrent obtained from the difference between light current and dark current, $P$ is the excitation power density, and $S$ is the active illuminated area.


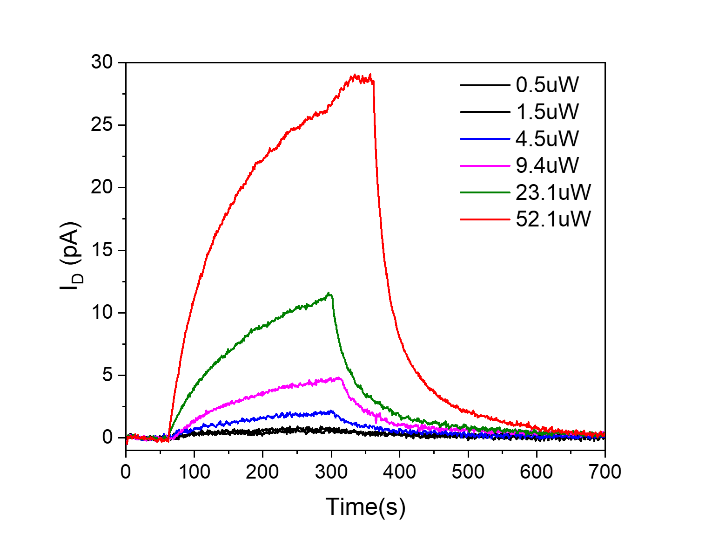


**Fig. S21. Time-dependent photoresponse of pristine 2H-MoS_2_ with different incident light power.**


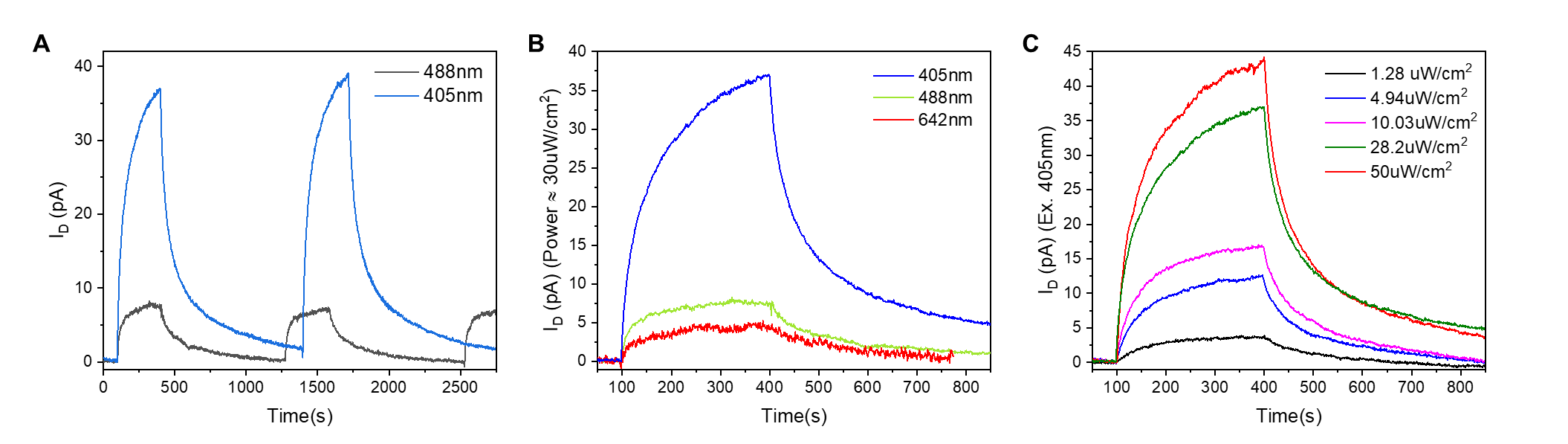


**Fig. S22.** **Time-dependent photoresponse of phase-engineering MoS_2_ with different incident wavelength and light power.** **A,** Repeatability at 488 nm, 405 nm excitation, **B,** Photoresponse with different incident light wavelength, **C,** Photoresponse with different incident light power.

As is demonstrated in Fig. S23, photorespinsivity ***R*** in phase-engineered MoS_2_ shows up to 10 times of enhancement at low power illumination compared to untreated 2H MoS_2_, reaching 85 A/W at V_DS_ = 1 V of bias. Comparing to literature-reported ***R*** (Table S3), our results indicate that a competitive R value could be achieved simply by phase engineering at the contact without gate modulation from the dielectric and high bias voltage.

**
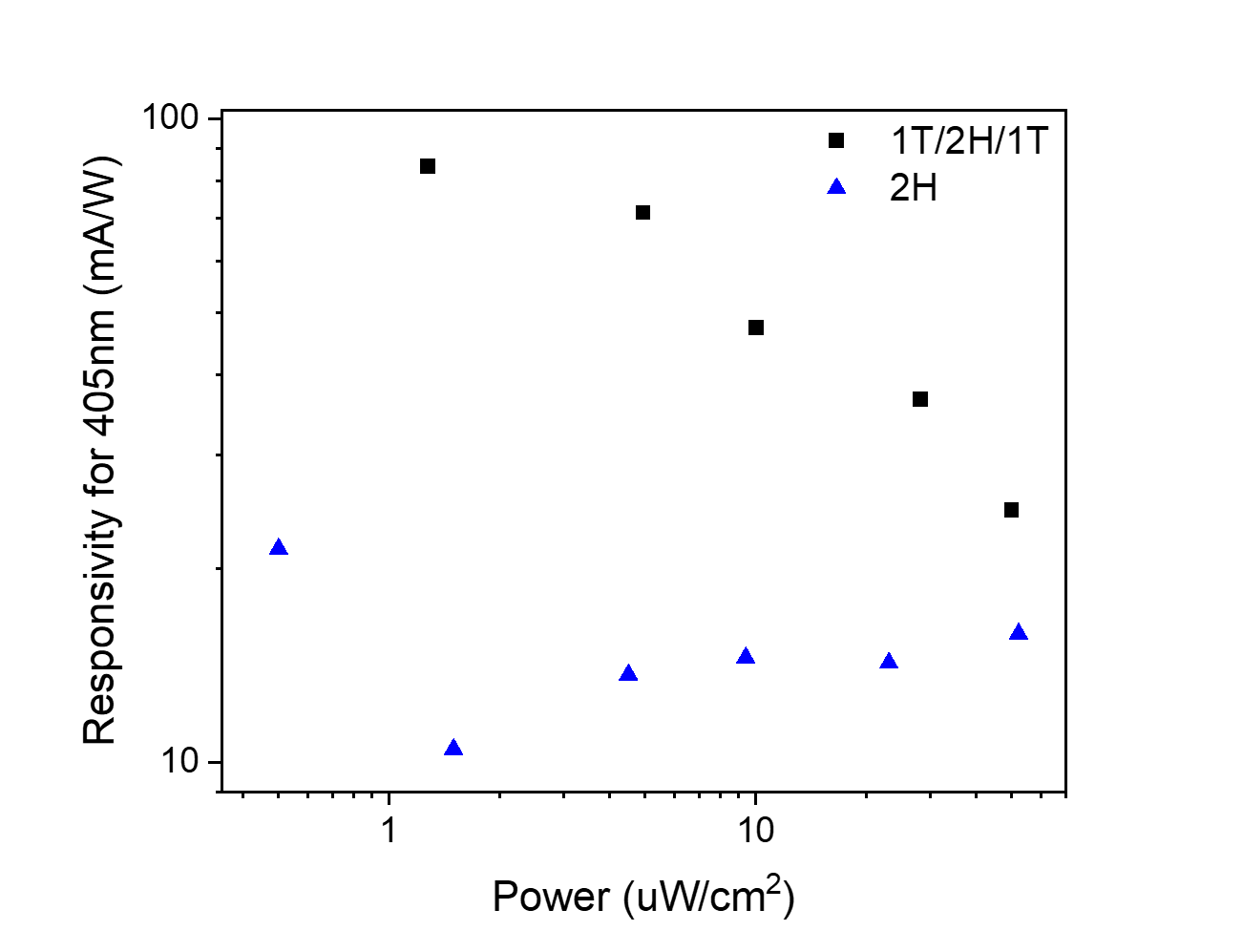
**

**Fig. S23.** **Responsivity comparison.**

| excitation | | Preparation Method | V_D_ (V) | Responsivity  (mA/W) | Ref. |
| --- | --- | --- | --- | --- | --- |
| Wavelength  (nm) | Power  (uW cm^-2^) |  |  |  |  |
| 405 | 1.28 | Phase-engineered exfoliated monolayer (1T/2H/1T)  (on microscopic cover glass  / Au electrode) | 1 | 84.35 | This  Work |
|  | 1.5 | Exfoliated monolayer  (on microscopic cover glass / Au electrode) | 1 | 10.49 |  |
| 550 | 80 μW | Exfoliated monolayer  (Ti/Au electrode) | 1 | 0.42 | ^53^ |
| 405 | ~24 μW | Exfoliated monolayer  (Au electrode, applied V_GS_) | 1~8 | 1.25 | ^49^ |
| 561 |  |  | 8 | 880  (V_GS_= -70 V) |  |
| 532 | 1.3 W | CVD-grown monolayer  (Ti/Au electrode, applied V_GS_) | 1 | 2200  (in Vacuum, V_GS_= -100 V) | ^50^ |
| 640 | ~3000  (30 W) | Suspended MoS_2_ | 0.5 | ~50000 | ^51^ |
| 530 | 2.35 mW | 2H-MoS_2_ on 1T-MoS_2_ | 10 | 37 | ^52^ |

**Table S3. Comparison for photodetector responsivity based on monolayer MoS_2_.**

**10. Contact property study using Fowler–Nordheim (FN) theory**

In Fig. 3B of the manuscript, we show how phase patterning enhances the photoresponsivity of a photodetector. This results from the 2H (semiconductor) to 1T (metal) phase engineering, which has shown its ability to reduce the contact resistance between MoS_2_ and metal.

The Schottky barrier for electron injection between MoS_2_ and the metal contact is one of the key problems in electronic applications for MoS_2_. The Schottky barrier height (Φ_B_) between MoS_2_ and metal has been reported to the level of few-hundreds meV^54–56^. Lowering the Schottky barrier for reducing the contact resistance, or switching Schottky behavior to Ohmic behavior enhances the transport properties of electronic devices using MoS_2_. It has been reported that phase engineering switches Schottky behavior to Ohmic-like behavior by renormalization of the band alignment and dramatically enhance the transport properties^20,21^. In our study, the photoresponsivity was enhanced by selectively inscribing a metallic 1T phase at the edge of the semiconducting 2H phase. Given the enhanced metal-semiconductor contact properties in same device geometry^20,21^, the enhanced photoresponsivity has been attributed to the switching the Schottky contact to Ohmic contact behavior by photo-redox phase engineering.

To provide further scientific supports, we conducted a detailed analysis of how electrons from metallic 1T phase is injected to the semiconducting 2H MoS_2_ channel. We analyzed the quantum transport behavior of our photodetector data using Fowler–Nordheim (FN) theory. FN theory suggests emission behavior of electrons from metallic emitters under electrical field, following the equation^57,58^ :

$$J_{FNT} \propto V^{2}exp[\frac{-8\pi\sqrt{2m*}\varphi_{B}^{\frac{3}{2}}d}{3hqV}]$$

(where $V$ : Bias voltage, $\varphi_{B}$ : tunneling barrier height, $m$ : effective electron mass, $h$: Planck constant, $q$ : elementary charge)

By plotting the J/V^2^ versus 1/V, we can obtain FN plot which suggest two regimes of field emissions^59^:

(1) Thermionic emission (TE) : Direct tunneling dominates (at large 1/V region, small bias voltage)

(2) FN tunneling dominates : Schottky barrier exists (at small 1/V region, large bias voltage)


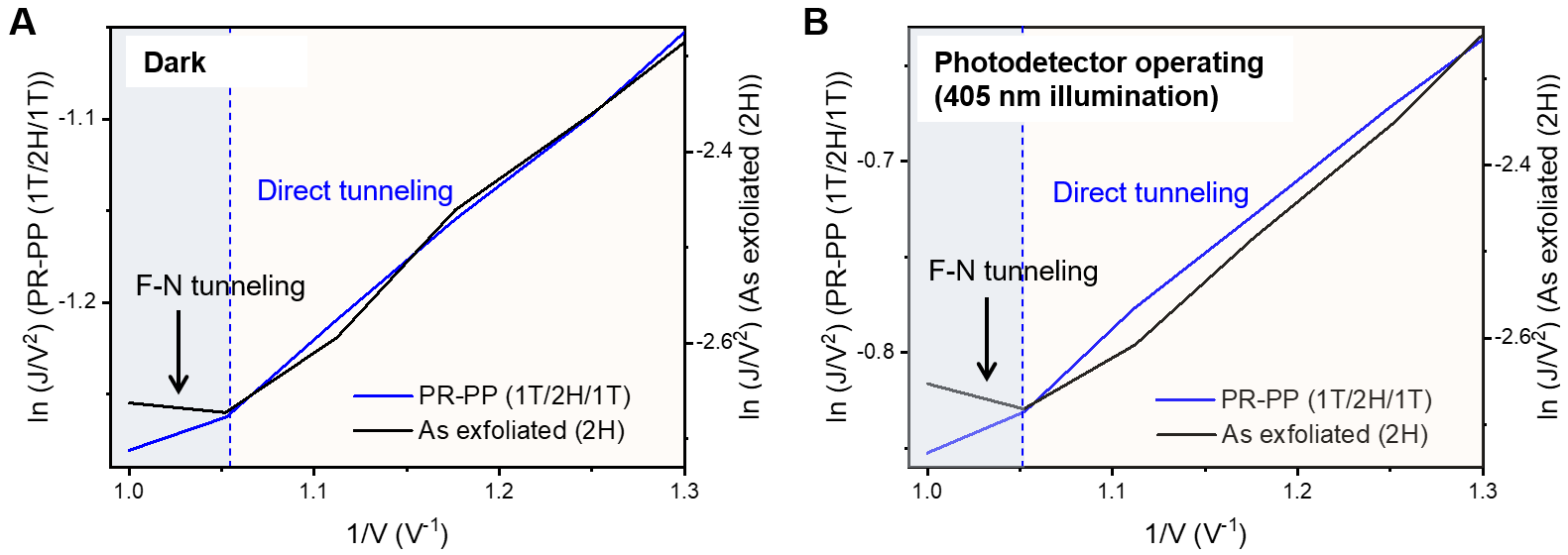


**Fig. S24. Fowler-Nordheim (FN) plot for monolayer MoS_2_ photodetector.** **A,** FN plot for photo-redox phase patterned (PR-PP), and As-exfoliated (2H) MoS_2_ photodetector measured in dark. **B,** FN plot for PR-PE, and As-exfoliated (2H) MoS_2_ photodetector during 405 nm illumination.

Fig. S24 A and B shows FN plot of photo-redox phase patterned (PR-PP), and no-patterned, as-exfoliated (2H) monolayer MoS_2_ photodetector under dark (A) and illuminated (B) conditions, respectively. Fig. S24 A shows the FN plot from the device made of 2H 1L-MoS_2_ (as-exfoliated, black curve) exhibits both direct tunneling (positive slope) and FN tunneling behavior (negative slope), while the photo-redox phase patterned (PR-PP) device showed only direct tunneling behavior (blue curve). This behavior remained when the photodetector was operated under 405 nm illuminations as shown in Fig. S24 B.

From here, we can conclude that the transport behavior in the PR-PP device switched from FN tunneling (i.e. a Schottky barrier exists) to direct tunneling at this voltage range by applying 2H to 1T phase engineering as illustrated in Fig. S25.


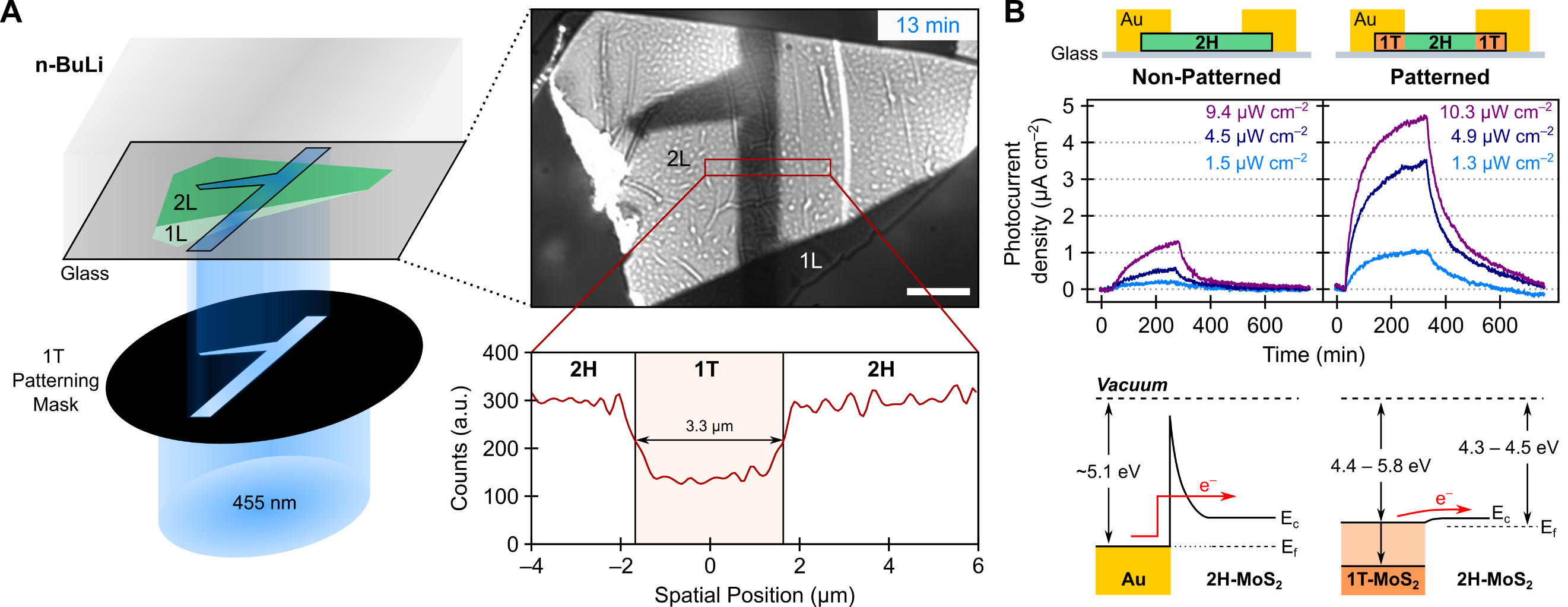


**Fig. S25. Schematics for electron tunnelling behavior for phase engineered MoS_2_ device, from Fowler-Nordheim (FN) analysis.** (E_c_ : conduction band energy level, E_f_: fermi energy level)

The work function of 2H MoS_2_ is around 4.3~ 4.5 eV depending on its surrounding environment^60,61^, and 1T MoS_2_ is varies from 4.4 eV to 5.8 eV depending on the adsorption of hydrogen functional groups^61^. Our analysis using Fowler–Nordheim (FN) tunneling model demonstrates the transition from a Schottky to an Ohmic behavior in the potential profile during photo-redox phase engineering. In literature, it has been reported that phase engineering switches Schottky behavior to Ohmic-like behavior by renormalization of the band alignment which dramatically enhances the transport properties^20,21^. Given the enhanced transistor performance in same device geometry^20,21^, the enhanced photoresponsivity in our study can also be attributed to Ohmic behavior.

**11. 1T-Phase MoS_2_ and WS_2_**


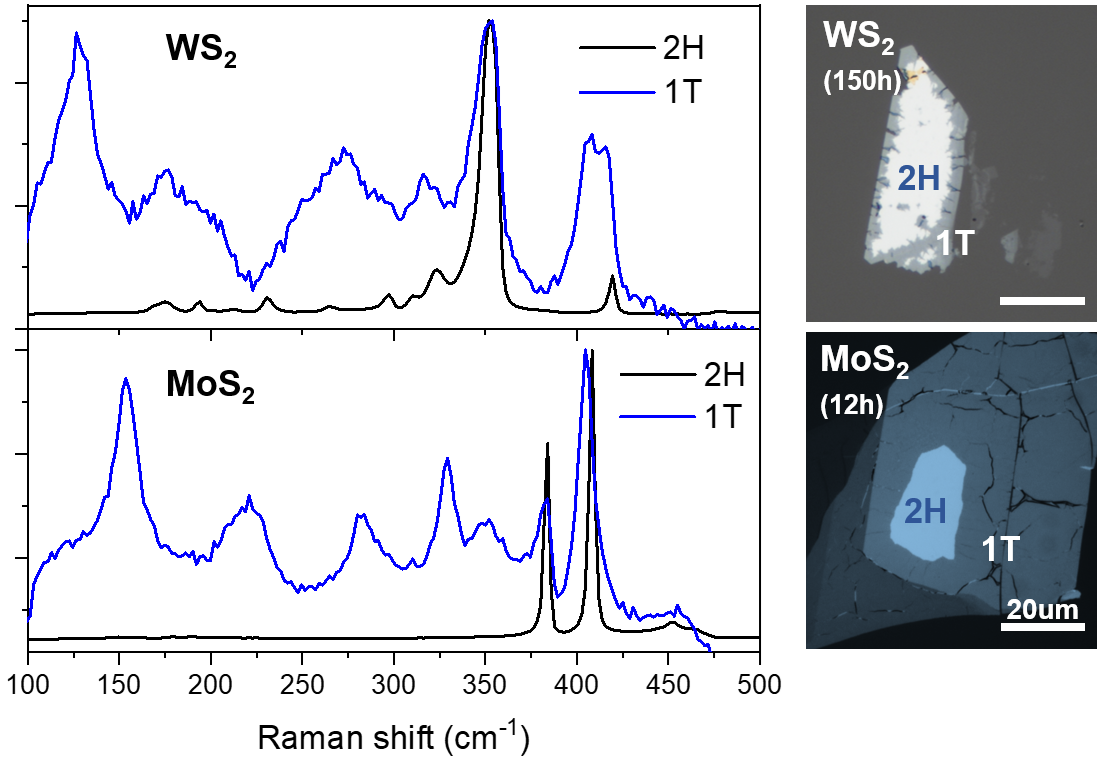


**Fig. S26. Raman spectra and optical image of 1T phase of MoS_2_ and WS_2_ using n-BuLi treatment in dark** (Scale bar = 20 μm).

In Fig. S26, the lithiation of thick-layer MoS_2_ and WS_2_ follows shrinking-core dynamics from the edge, as previously reported^15^. The outer-ring region for both MoS_2_ and WS_2_ showed J-peaks of Raman similar to synthesized 1T-WS_2_, which indicates the phase changed to 1T ^62–64^.

However, this spontaneous phase transition for WS_2_ in room temperature is very slow (150 h) and rarely observed (very low yield). So, spontaneous phase transition reaction with **n-BuLi** in room temperature is much less favorable to WS_2_ than MoS_2_.

**12. Phase transition and conversion by above-gap illumination(455nm) on 2H-TMDs**


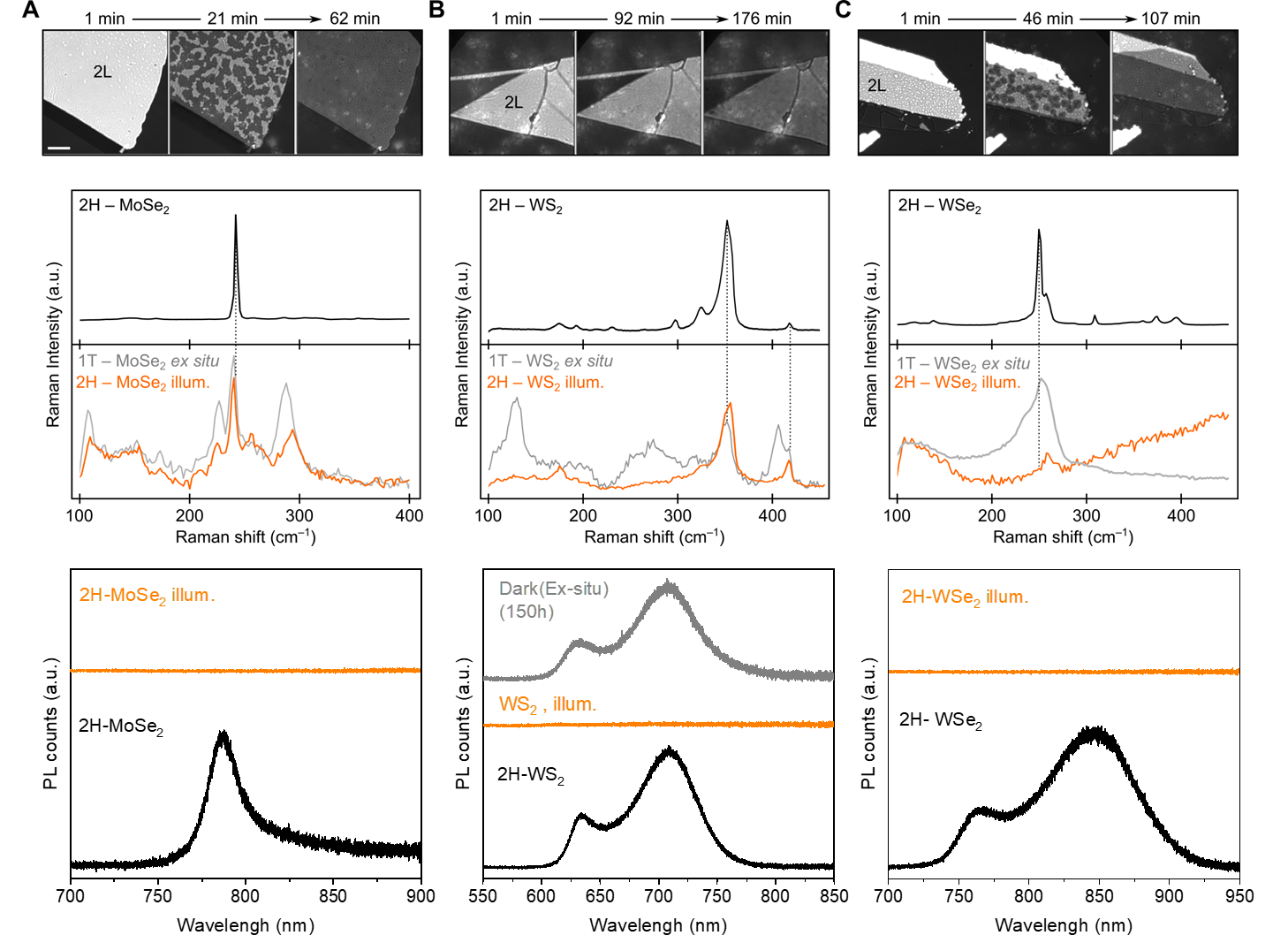


**Fig. S27.** **Snapshot of phase transition dynamics measured in RICN at different time and steady-state PL and Raman spectra of as-exfoliated, illuminated, and ex-situ(48h n-BuLi treatment in dark) bilayer TMDs.** **A,** MoSe_2_, **B,** WS_2_, **C,** WSe_2_ (Scale bar = 5 μm).

In Fig. S27 A, the RICM image of MoSe_2_ shows surface darkening followed by wave front similar to the MoS_2_ (Fig. 2 in main paper). The wavefront arise not only from the edges but also from the inside of the flake, which could be due to the internal structural strain or defect. MoSe_2_ changed the phase to 1T, as showing the Raman peaks at J_1_ (106.4 cm^-1^), J_2_ (150.7 cm^-1^), J_3_ (221.4 cm^-1^), E^1^_2g_ (289.4 cm^-1^), at both illuminated and non-illuminated (dark, *ex-situ*) condition^30,65,66^. In PL signal, the illuminated sample (orange line)showed disappearance of PL as MoS_2_. Therefore, the photo-redox process worked for phase transition from 2H to 1T phase as similar to MoS_2_.

For WS_2_, the overall-darkening on the flake happened (RICM image in Fig 4A), and it did not show a 1T signal from Raman but only 2H signal with extremely high signal-to noise ratio (compare grey and orange curve at Fig. S27 B). At the same time, the illuminated sample only showed disappearance of PL while the PL stays similar up to 150 h at dark. It describes the light driven reaction triggers not only phase transition but also conversion reaction. Also, the existence of PL shows a lower possibility of spontaneous phase transition of WS_2_ at dark than MoS_2_.

In Fig. S27 C, RICM images of WSe_2_ display the surface darkening followed by two wave front moving at very closely. And after the lithiation or dark experiment, no 1T signature found by Raman, with disappearance of PL^67^. In Fig. S30 B, the electrochemical potential for WS_2_ has two plateau at very close voltage range and WSe_2_ has single plateau. The same redox potential can be measured by CV (Fig. S29). The very close, or overlapped electrochemical potential WX_2_ (X=S, Se) could be related to the direct conversion reaction (irreversible decomposition to W and Li_X_S or Li_X_Se) of WX_2_ at both electrochemical, and chemical lithiation with above-gap illumination. Although we get the similar results of conversion from WX_2_, we could see the two wave front moving at very closely only at WSe_2_. We hypothesized this is mainly due to the most ionic nature of sulphides in chalcogen atoms (with the sequence being S>Se>Te)^68^.

**13. Electrochemical interpretation of phase transition**

_
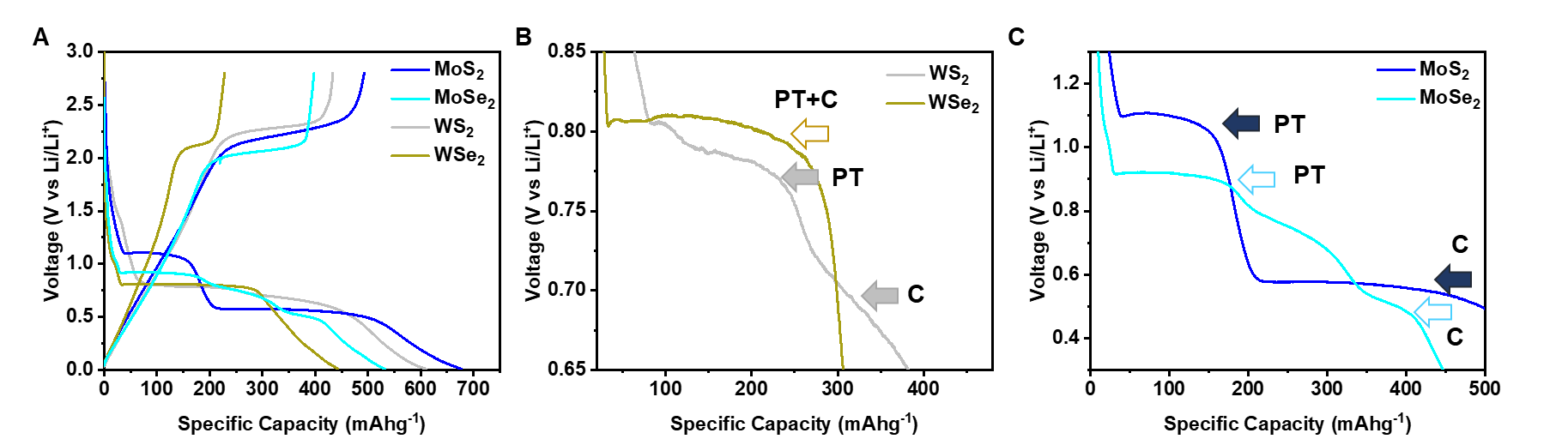
_

**Fig. S28. Extended Data Fig. 5 The** **Galvano static charge-discharge curve. A,** Galvano static charge-discharge curve of MoS_2_, MoSe_2_, WS_2_, and WSe_2_ (Voltage range : 0 – 3.0 V vs Li/Li^+^) **B,** Discharge curve for WS_2_, WSe_2_ (in voltage range of 0.65 – 0.85 V vs Li/Li+ (Magnified from (**A**)) **C,** Discharge curve for MoS_2_, MoSe_2_  (in voltage range of 0.3 – 1.3 V vs Li/Li+ (Magnified from (**A**)).(PT = 2H to 1T Phase transition, C = Conversion reaction.).


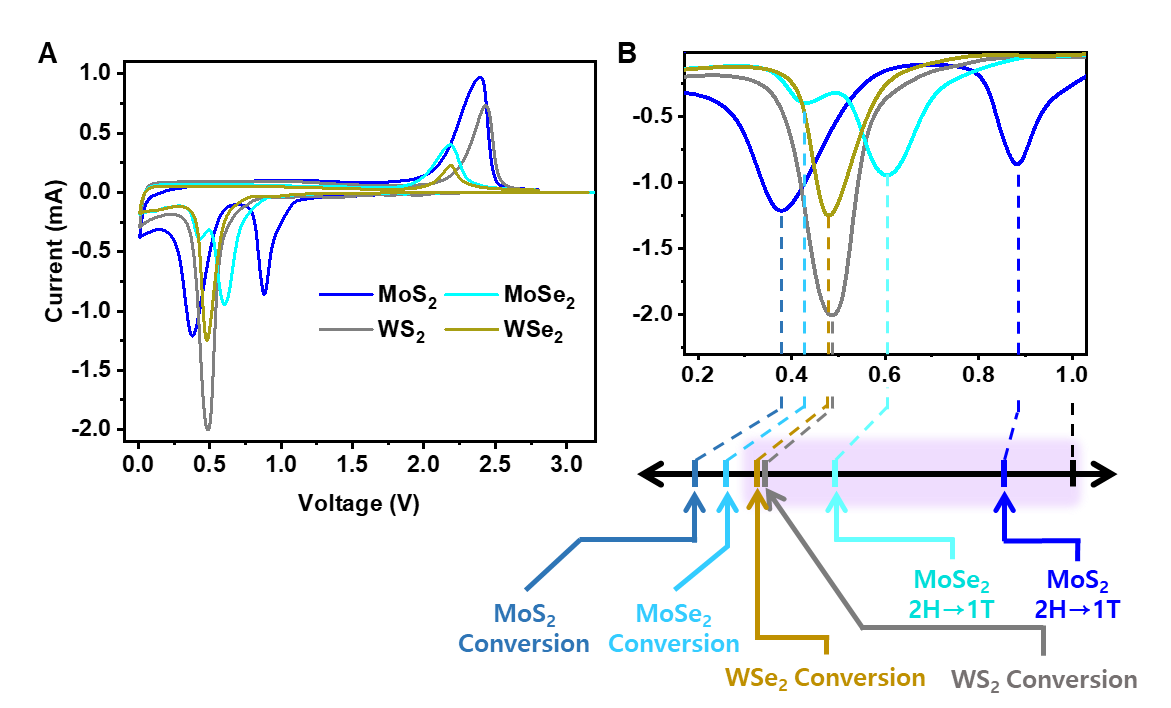


**Fig. S29. Cyclic voltammetry(CV) curve. A,** CV graph of MoS_2_, MoSe_2_, WS_2_, and WSe_2_ **B,** Magnified from A for the volage range of 0.2~1.0 V and redox potential peak diagram.

**14. Steady-state PL spectra from the illumination edge of WX_2_ (X=S, Se)**


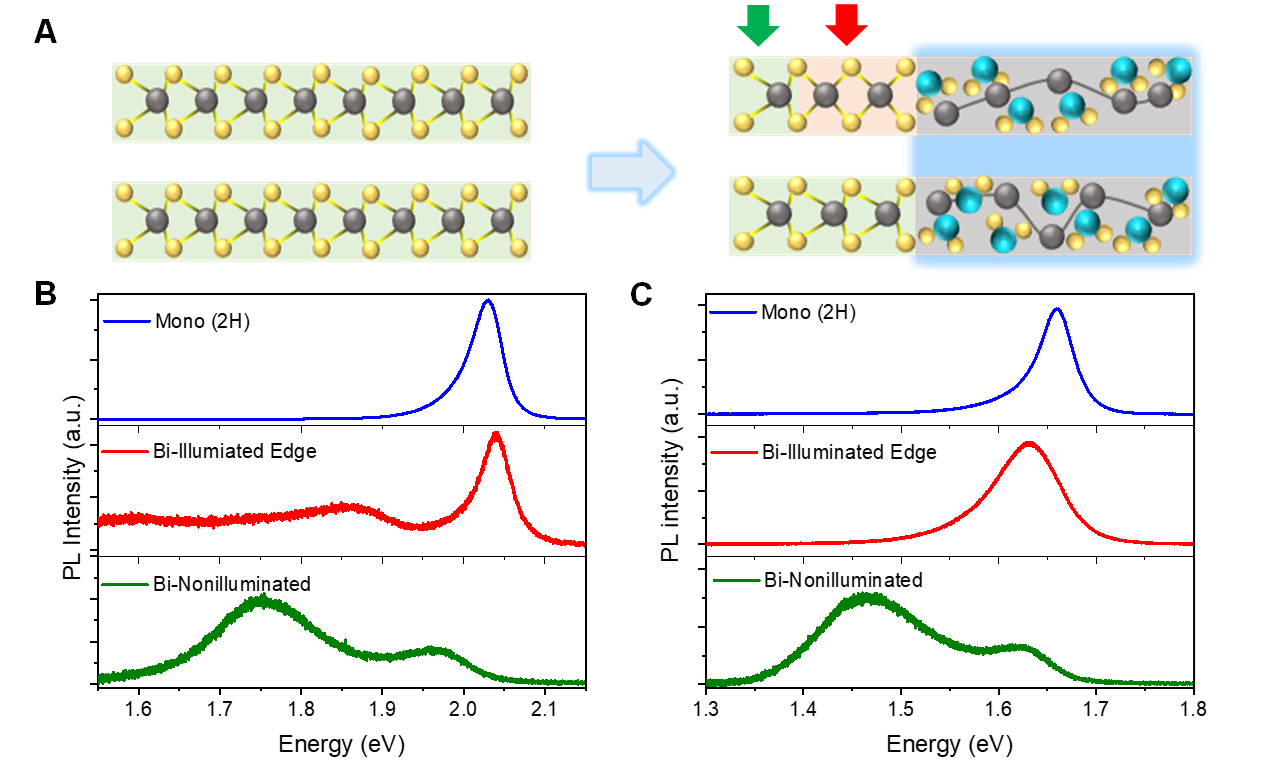


**Fig. S30.** **Ex-situ PL from the non-illuminated and illumination edge for bilayer WX_2_ (S = S, Se). A,** Schematics of initial dynamics for phase transition. **B,** PL of Nonilluminated bilayer (exposed to **n-BuLi** in dark), Illuminated edge, and exfoliated monolayer of WS_2_ **C,** PL of Nonilluminated bilayer, Illuminated edge, and exfoliated monolayer of WSe_2_.

Fig. S30 shows the PL data from the illumination-edge of bilayer WS_2_, and WSe_2_. Compare to the MoS_2_, the 455 nm illuminated samples show a slightly different color at the illumination edge. This spot exhibits monolayer-like PL, while the illuminated area has no PL (conversion reaction already happened) and the non-illuminated area still shows the PL signal of the bi-layer (2H phase, see also Fig. S19).

We postulate that when light activates both the phase transition and the conversion reaction, the illumination alters only the phase of the first layer and resulting in a strong monolayer-like PL. Compared to MoS_2_, which shows monolayer-like PL signal for the whole non-illuminated bilayer (Fig. S19) by the weakening of interlayer interaction, this localized monolayer-PL signal from WX_2_ (X=S, Se) explains different initial dynamics close the solid-solution-like reaction.

**15. Reversibility of 1T-MoS_2_**


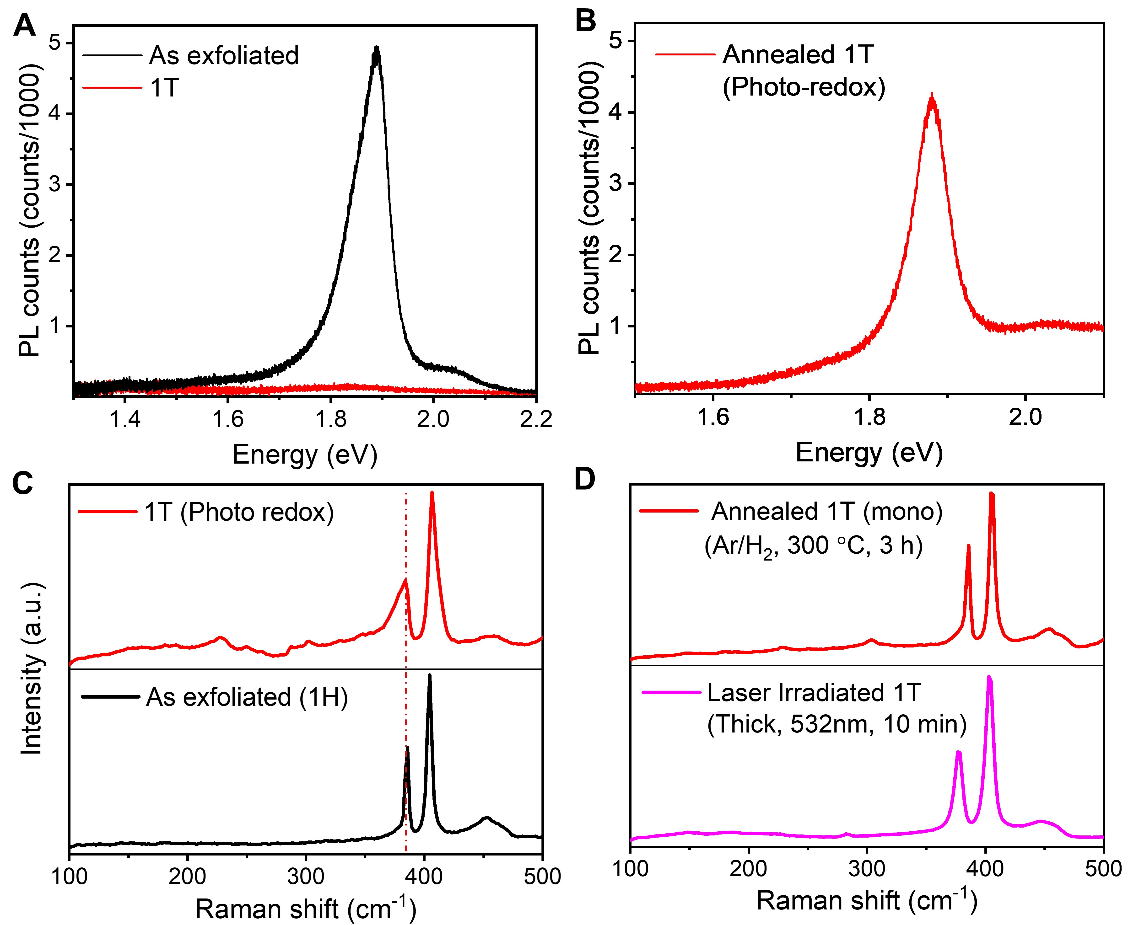


**Fig. S31. Ex-situ Raman and PL analysis for monolayer MoS_2_. A,** PL of as exfoliated and photo-redox phase patterned monolayer 1T MoS_2_. **B,** PL of photo-redox phase patterned and thermally annealed 1T. **C,** Raman spectra of as exfoliated (1H, bottom) and photo-redox phase patterned 1T MoS_2_ (top). **D,** Raman spectra of annealed 1T (photo-redox phase patterned and annealed sample) (red, top), and laser-irradiated thick 1T MoS_2_ sample (pink, bottom).

The phase transition of MoS_2_ is reversible. The 1T MoS_2_ phase can be converted back to 2H phase by both thermal annealing and laser irradiation^22,33^. In Fig. S31, the photo-redox patterned monolayer is reversible and converts back to 2H phase during thermal annealing (in Ar/H_2_ atmosphere, 300 ℃, 3 hours). This confirmed by PL restoration (B), and Raman spectra (D, top). Here, the E_2g_ peak gets narrower and blue-shifted as the phase restores back to 2H phase by thermal annealing.

The reversibility was also confirmed in thick exfoliated sample (more than 10 layers) using strong laser irradiation. We irradiated a thick 1T sample using a confocal Raman setup (~10 min, 532 nm, 500 mW, focused on diffraction-limited spot (NA = 0.85)). The Raman spectra from the thick 1T MoS_2_ return to their 2H phase, as the bottom graph of D (pink line). This shows that the 1T phase MoS_2_ made by either conventional process (48h n-BuLi treatment) and photo-redox engineering process is reversible.

The reversibility also can be found in the Fig. S8, that shows thermal annealing restores the PL signal and shifts E_2g_ back to original 2H-phase position in monolayer.

**16. Novel organolithiation agent : Polycyclic Aromatic Hydrocarbons (PAHs)-Li System**

In Fig. 5, we have provided comprehensive insights into phase engineering by establishing a correlation with *in-situ* optical data and electrochemical potential. We also emphasize the significant role of chemical potential in this process. For MoS_2_, the electrochemical potential associated with the phase transition is around 1.1 V (versus Li/Li^+^), and another lower potential appears around 0.57 V (versus Li/Li^+^), assigned to conversion or amorphization via irreversible decomposition. Consequently, the redox potential of the chemical utilized for the phase transition must be located within these two potential. It aligns well with the spontaneous reaction of n-butyllithium (n-BuLi). The n-BuLi has a redox potential of 1 V (versus Li/Li^+^), initiating phase transition but do not do cause a further conversion process. Based on this understanding and a desire to eliminate pyrophoric n-BuLi, we synthesized novel organolithiation agents to phase engineer TMD materials.

Table S4 shows chemical formula, structure, and redox potential of different polycyclic aromatic hydrocarbons (PAHs) ^69–71^. Although the use of different electrolytes, concentration and measurement properties varies the absolute redox potential value, we can identify a trend of decreasing the redox potential as the number of benzene rings decreases. For phase engineering of MoS_2_ by lithiation, we have replaced the carbon component (‘n-butyl’ from ‘n-butyllithium’) to PAHs having appropriate redox potential, between 1.1 V and 0.57 V (versus Li/Li^+^).

| Name | Chemical  formula | Chemical  structure | Redox potential  (Approx. versus Li/Li^+^) |
| --- | --- | --- | --- |
| Tetracene | C_18_H_12_ |  | 1.44 V |
| **Perylene** | C_20_H_12_ | 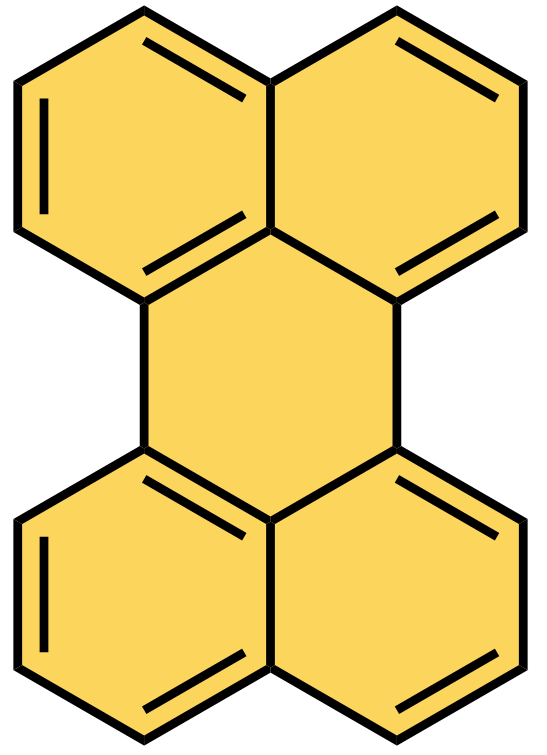 | 1.19 ~ 1.35 V |
| **Anthracene** | C_14_H_10_ |  | 0.91 ~ 1.03 V |
| **Pyrene** | C_16_H_10_ | 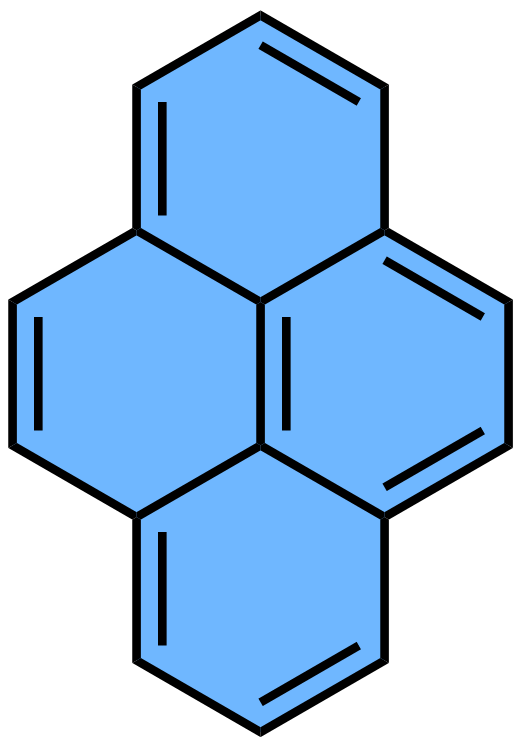 | 0.73 ~ 0.93 V |
| Phenanthrene | C_14_H_10_ | 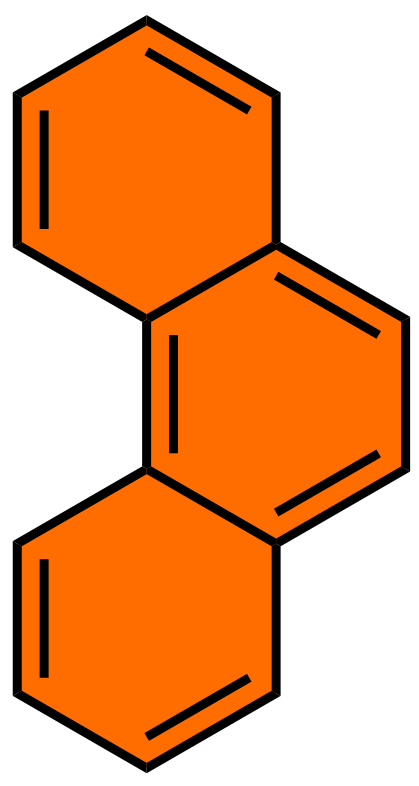 | 0.31 ~ 0.54 V |
| **Naphthalene** | C_10_H_8_ |  | 0.26 ~ 0.46 V |
| Biphenyl | C_12_H_10_ | 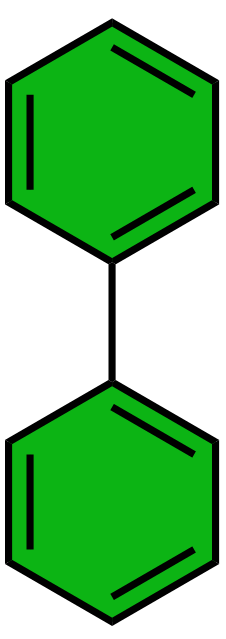 | 0.17 ~ 0.41 V |

**Table S4. The chemical formula, structure and redox potential (versus Li/Li^+^) of different polycyclic aromatic hydrocarbons (PAHs)**

From this table, we selected Naphthalene, Anthracene, Pyrene and Perylene as candidate for studying PAHs-Li system on MoS_2_. We used anhydrous tetrahydrofuran (THF) as the organic solvent^72,73^. PAHs dissolved in anhydrous tetrahydrofuran (THF) solvent in its maximum solubility. Lithium metal (lithium granular) added in PAHs-THF solvent with the stoichiometric ratio of PAH : Li is the same.


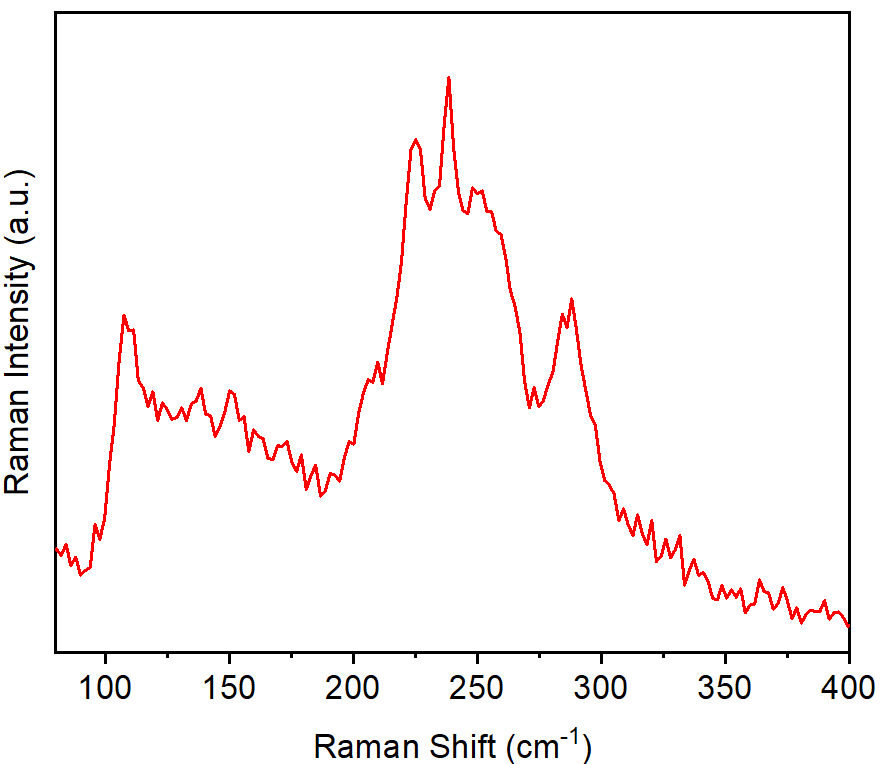


**Fig. S32. Raman spectra of MoSe_2_ treated with Anthracene-Li systems.**

Fig. 5A in the main manuscript shows the Raman spectra measured for thick layer of MoS_2_ treated with each PAH-Li. We mechanically exfoliated MoS_2_ on glass substrate and treated each PAH-Li agent for 1 hour. We washed the sample using THF and measured Raman using thick-lithiated 1T MoS_2_. All sample used here are thick samples (more than 4-5 layers by optical contrast at exfoliation). In Fig. 5A, we can clearly see the 1T signatures of a shifted and broadened E_2g_ peak, with the emergence of J peaks from Pyrene-Li (purple), and Anthracene-Li (green) treated MoS_2_. Both PAHs have redox potentials between 1.13 V and 0.57 V and expected to drive phase transition reaction but not further decomposition (conversion, amorphization). Interestingly, compared to n-BuLi which usually takes around 48 hours for the reaction (blue line), the reaction using PAH-Li takes less than 1 hour to complete in thick flakes. The use of Naphthalene-Li (orange) shows the material goes down to conversion reaction that has no Raman signature of either 2H, or 1T MoS_2_. This results also aligns with literature used Naphthalene-Li and Pyrene-Li in 1,2-Dimethoxyethane (DME) solvent^70^. Conversely, the THF-Anthracene without lithium, and Perylene-Li(grey line) chemicals were unable to drive the reaction.

In Fig. S32, we used the Anthracene-Li system on exfoliated, few-layered MoSe_2_. In Fig. S28, MoSe_2_ shows two potentials of 0.9 V, and 0.5 V. Therefore, we used anthracene-Li system for 1 hour. The Raman signature shows 1T signature as similar to n-BuLi treated MoSe_2_ (Fig S27). This shows that Anthracene-Li system is working similar to n-BuLi, albeit with faster kinetics.


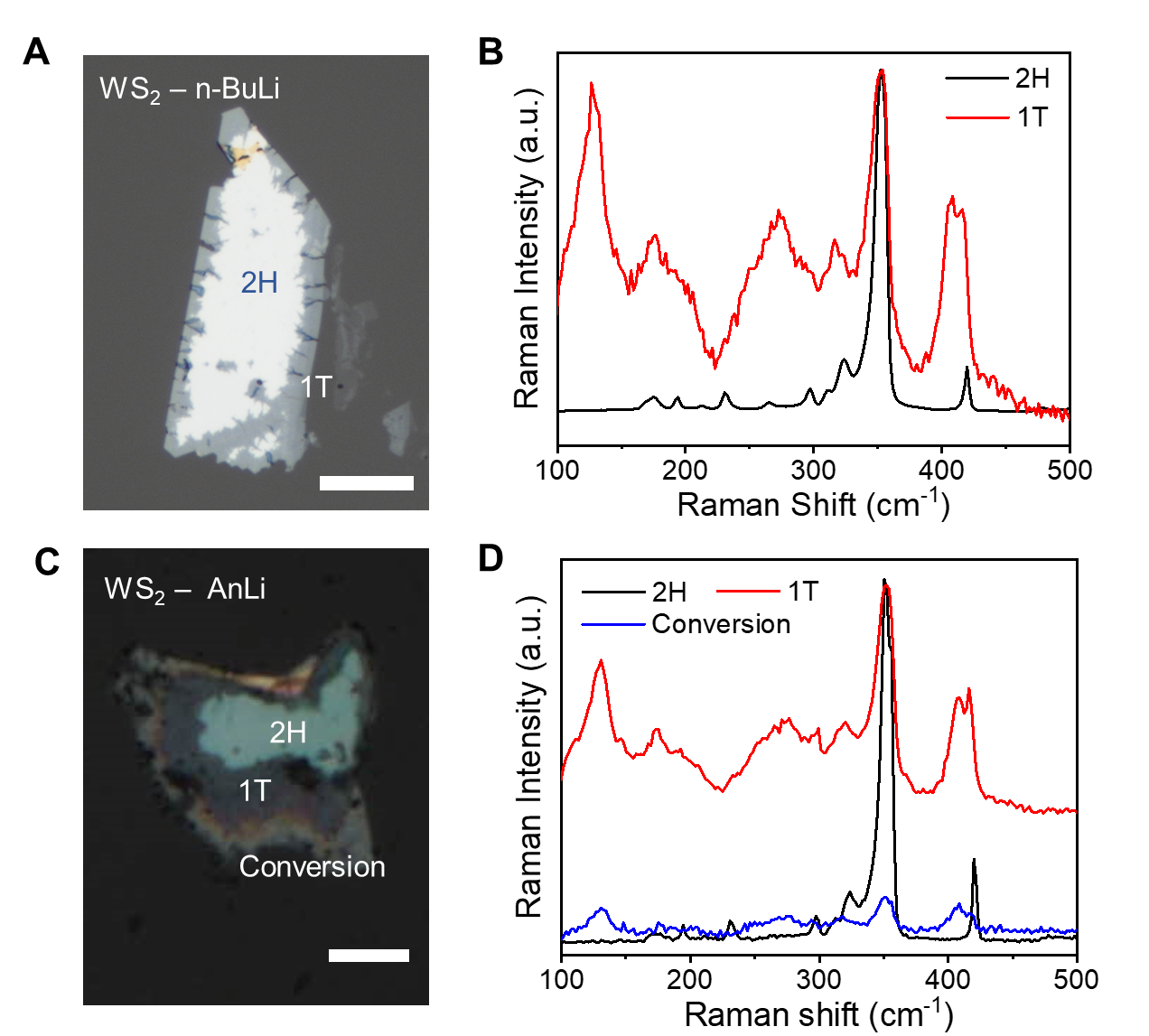


**Fig. S33. Optical image and Raman spectrum of n-BuLi treated, and Anthracene-Li (AnLi) treated WS_2_.** **A,** Optical image of n-BuLi treated WS_2_. Shrinking core-type phase front is observed. **B,** Raman spectra of 1T WS_2_ (red, from outer ring region in A) and 2H WS_2_ (black, from inner region in A) **C,** Optical image of AnLi treated WS_2_. Two wave fronts are observed. **D,** Raman spectra of 1T WS_2_ (red, middle ring region in **C**) and 2H WS_2_ (black, from inner region in **C**), and converged region (blue, out most region in **C**) (Scale bar for A, C = 20 μm) (**A**, **B** adapted from Fig. S26)

We further explored the AnLi system for phase transformation in WS_2_. We used exfoliated WS_2_ on glass substrate and treated AnLi without light in same method as MoS_2_ (in Fig. S34 A, C), or MoSe_2_ (in Fig. S32). As explained in section 11, WS_2_ shows single phase front in n-BuLi at the dark with a shrinking-core fashion inside the flake (Fig. S 33 A). In Fig. S33 B, Raman spectra confirm that the outer region changed to the 1T phase (red), while the core remained 2H (black), with no decomposition products detected.

In Fig. S33 C-D the use of the anthracene-Lithium driven the reaction further to conversion (decomposition). Optically, in Fig. S33 C, we can identify three concentrically located phases which can be assigned by Raman spectroscopy to 2H in the center, a converted phase (outer ring, blue in D), and an intermediate 1T phase (middle ring region in C and red graph in D). This demonstrates that lithiation of WS_2_ follows a two-step process and direct conversion is not the most preferred pathway. The use of AnLi in WS_2_ shows both the accelerated phase transition speed from lower redox potential and the spontaneous occurrence of phase transition and decomposition reactions in order, which is related to their closely located electrochemical potential.

**17. Photo-redox phase engineering with PAHs-Li System**


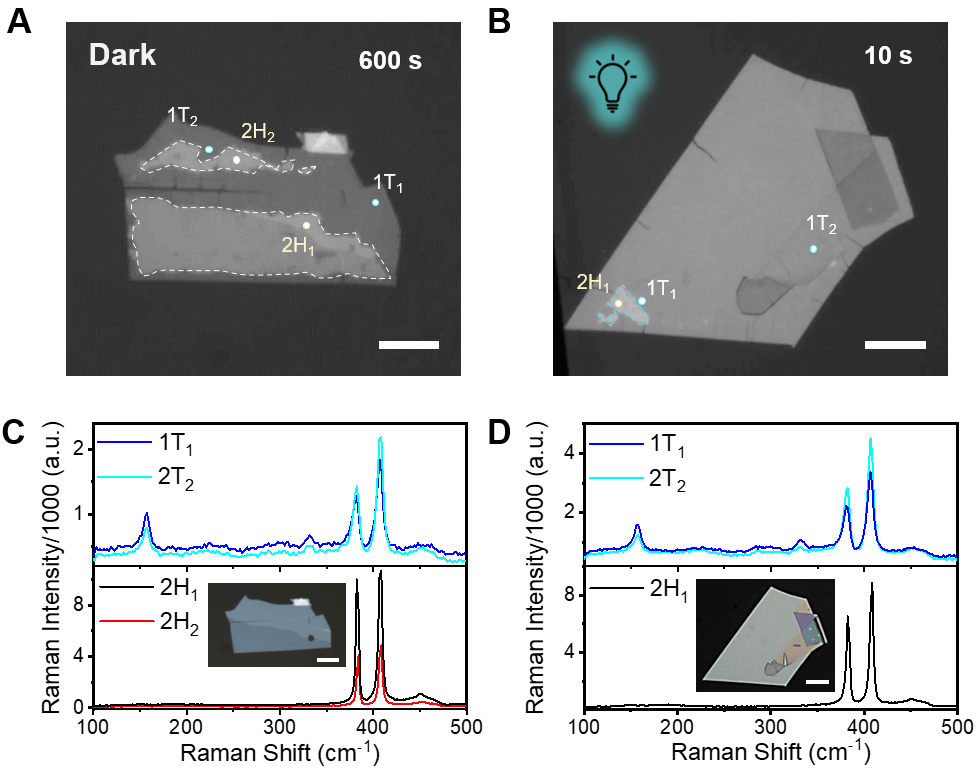


**Fig. S34. Photo-redox phase transition using Anthracene-Li system. A-B,** adapted from **Fig. 5 B- C** with designating the point for *ex-situ* Raman measurement. **C,** Raman spectra measured from Sample in **A** from each spot. The inset image of **C** shows the as-exfoliated image of the flake measured in **A, C. D,** Raman spectra measured from Sample in **B**. The inset of D image shows the as-exfoliated image of the flake measured in **B, D.** (scale bar for all figure = 5 μm)

We further investigated the photo-redox process when using anthracene-Li (AnLi). We exfoliated a thick MoS_2_ (>>10 layers) on glass substrate and placed it in an empty glass vial under ambient conditions before filling it with the anthracene-Li solution. The as-exfoliated each flake is shows in the inset image of Fig. S34 C and D. From the optical contrast of each flake, we figure out the thickness of sample used for Fig. S34 B, D is more thicker than sample for Fig. S34 A, C. One vial (with sample image in the bottom of Fig. S34 C) was allowed to react with the solution for 10 min without any light, while another vial (with sample image in the bottom of Fig. S39 D) was exposed to 445 nm LED illumination (SOLIS-1D, power density = 130 mW cm^-2^) for 10 seconds. We washed both samples and monitored the reaction using optical images of each thick flakes *ex-situ*.

In Fig. S34 A and B, we observe clear wavefronts as we observed with n-BuLi treated thick-MoS_2_^15^. From the wavefront shape, we can roughly calculated a phase front speed for comparison. The wave front speed of flake A (dark) showed ~ 5 nm s^-1^, while flake B (illuminated) showed a markedly enhanced speed of ~ 5 μm s^-1^. These front speeds are significantly faster than what we could achieve using n-BuLi with illumination and enable the process even in thick flakes, where the combination of redox matching and above-gap illumination accelerates the reaction significantly. Fig. S34 C, D shows the Raman spectra of each point in Fig. S34 A, B. The inner area (spot 2H_1-2_ in A and spot 2H_1_ in B) remains 2H phase (red, black curve), while the outer ring region (1T_1-2_ in A, B) changed to 1T phase (blue, cyan curve).

In conclusion, here we synthesize novel redox-based organolithiation agent (PAH-Li) which is easy to use, safe (non-pyrophoric) and perform rapid phase engineering to MoS_2_ and MoSe_2_. This can replace n-BuLi and could make whole process faster and safer and greener. Furthermore, the photo-redox process works with PAH-Li system and accelerates the reaction. When using both photo-redox process with PAH-Li, the whole reaction accelerated 5-6 orders of magnitude than conventional n-BuLi treatment.

References

1. Born, M. et al. Principles of Optics: Electromagnetic Theory of Propagation, Interference and Diffraction of Light. (Cambridge University Press, 1999).

2. Mak, K. F., Lee, C., Hone, J., Shan, J. & Heinz, T. F. Atomically thin MoS_2_: A new direct-gap semiconductor. *Phys. Rev. Lett.* **105**, (2010).

3. Tonndorf, P. et al. Photoluminescence emission and Raman response of monolayer MoS_2_, MoSe_2_, and WSe_2_. *Opt. Express* **21**, 4908–4916 (2013).

4. Zhao, W. et al. Evolution of Electronic Structure in Atomically Thin Sheets of WS_2_ and WSe_2_. *ACS Nano* **7**, 791–797 (2013).

5. Peimyoo, N. et al. Thermal conductivity determination of suspended mono- and bilayer WS_2_ by Raman spectroscopy. *Nano Res.* **8**, 1210–1221 (2015).

6. Zeng, H. et al. Optical signature of symmetry variations and spin-valley coupling in atomically thin tungsten dichalcogenides. *Sci. Rep.* **3**, 2–6 (2013).

7. Bissett, M. A., Worrall, S. D., Kinloch, I. A. & Dryfe, R. A. W. Comparison of Two-Dimensional Transition Metal Dichalcogenides for Electrochemical Supercapacitors. *Electrochem. Acta* **201**, 30–37 (2016).

8. Li, H. et al. From bulk to monolayer MoS_2_: Evolution of Raman scattering. *Adv. Funct. Mater.* **22**, 1385–1390 (2012).

9. Calandra, M. Chemically exfoliated single-layer MoS_2_: Stability, lattice dynamics, and catalytic adsorption from first principles. *Phys. Rev. B* **88**, (2013).

10. Yang, D., Sandoval Jimenez, S., Divigalpitiya W. M. R., Irwin, J. C. & Frindt, R. F. Structure of single-molecular-layer MoS_2_. *Phys. Rev. B* **43**, 53–56 (1991).

11. Jiménez Sandoval, S., Yang, D., Frindt, R. F. & Irwin, J. C. Raman study and lattice dynamics of single molecular layers of MoS_2_. *Phys. Rev. B* **44**, 3955–3962 (1991).

12. Xia, J. et al. Phase evolution of lithium intercalation dynamics in 2H-MoS_2_. *Nanoscale* **9**, 7533–7540 (2017).

13. Attanayake, N. H. et al. Effect of Intercalated Metals on the Electrocatalytic Activity of 1T-MoS_2_ for the Hydrogen Evolution Reaction. *ACS Energy Lett*. **3**, 7–13 (2018).

14. Jiao, Y. et al. Metallic MoS_2_ for High Performance Energy Storage and Energy Conversion. *Small* **14**, (2018).

15. Xiong, F. et al. Li Intercalation in MoS_2_: In Situ Observation of Its Dynamics and Tuning Optical and Electrical Properties. *Nano Lett.* **15**, 6777–6784 (2015).

16. Tan, S. J. R. et al. Chemical Stabilization of 1T′ Phase Transition Metal Dichalcogenides with Giant Optical Kerr Nonlinearity. *J. Am. Chem. Soc.* **139**, 2504–2511 (2017).

17. Ng, H. K. et al. Effects of Structural Phase Transition on Thermoelectric Performance in Lithium-Intercalated Molybdenum Disulfide (LixMoS_2_). *ACS Appl. Mater. Interfaces* **11**, 12184–12189 (2019).

18. Tan, S. J. R. et al. Temperature- and Phase-Dependent Phonon Renormalization in 1T’-MoS_2_. *ACS Nano* **12**, 5051–5058 (2018).

19. Voiry, D. et al. The role of electronic coupling between substrate and 2D MoS_2_ nanosheets in electrocatalytic production of hydrogen. *Nat. Mater.* **15**, 1003–1009 (2016).

20. Kappera, R. et al. Metallic 1T phase source/drain electrodes for field effect transistors from chemical vapor deposited MoS_2_. *APL Mater*. **2**, (2014).

21. Kappera, R. et al. Phase-engineered low-resistance contacts for ultrathin MoS_2_ transistors. *Nat. Mater.* **13**, 1128–1134 (2014).

22. Eda, G. et al. Photoluminescence from chemically exfoliated MoS_2_. *Nano Lett.* **11**, 5111–5116 (2011).

23. Pondick, J. V. et al. Thickness-dependent phase transition kinetics in lithium-intercalated MoS_2_. *2D Mater.* **9**, (2022).

24. El Garah, M. et al. MoS_2_ nanosheets via electrochemical lithium-ion intercalation under ambient conditions. *FlatChem* **9**, 33–39 (2018).

25. Yu, Y. et al. High phase-purity 1T′-MoS_2_- and 1T′-MoSe_2_-layered crystals. *Nat. Chem.* **10**, 638–643 (2018).

26. Voiry, D. et al. Conducting MoS_2_ nanosheets as catalysts for hydrogen evolution reaction. *Nano Lett.* **13**, 6222–6227 (2013).

27. Li, Z. et al. Lithiated metallic molybdenum disulfide nanosheets for high-performance lithium–sulfur batteries. *Nat. Energy* **8**, 84–93 (2023).

28. Lukowski, M. A. et al. Enhanced hydrogen evolution catalysis from chemically exfoliated metallic MoS_2_ nanosheets. *J. Am. Chem. Soc.* **135**, 10274–10277 (2013).

29. Ding, Q. et al. Efficient photoelectrochemical hydrogen generation using heterostructures of Si and chemically exfoliated metallic MoS_2_. *J. Am. Chem. Soc.* **136**, 8504–8507 (2014).

30. Gupta, U. et al. Characterization of few-layer 1T-MoSe_2_ and its superior performance in the visible-light induced hydrogen evolution reaction. *APL Mater*. **2**, (2014).

31. Wang, Y., Sofer, Z., Luxa, J. & Pumera, M. Lithium Exfoliated Vanadium Dichalcogenides (VS_2_, VSe_2_, VTe_2_) Exhibit Dramatically Different Properties from Their Bulk Counterparts. *Adv. Mater. Interfaces* **3**, 1–8 (2016).

32. Zhang, X. et al. Two-dimensional MoS_2_-enabled flexible rectenna for Wi-Fi-band wireless energy harvesting. *Nature* **566**, 368–372 (2019).

33. Guo, Y. et al. Probing the Dynamics of the Metallic-to-Semiconducting Structural Phase Transformation in MoS_2_ Crystals. *Nano Lett.* **15**, 5081–5088 (2015).

34. Zhu, B., Chen, X. & Cui, X. Exciton binding energy of monolayer WS_2_. *Sci. Rep.* **5**, (2015).

35. Wang, L. et al. Slow cooling and efficient extraction of C-exciton hot carriers in MoS_2_ monolayer. *Nat. Commun.* **8**, (2017).

36. Kim, H. J., Yun, Y. J., Yi, S. N., Chang, S. K. & Ha, D. H. Changes in the Photoluminescence of Monolayer and Bilayer Molybdenum Disulfide during Laser Irradiation*. ACS Omega* **5**, 7903–7909 (2020).

37. Hu, L., Shan, X., Wu, Y., Zhao, J. & Lu, X. Laser thinning and patterning of MoS_2_ with layer-by-layer precision. *Sci. Rep.* **7**, 1–9 (2017).

38. Rho, Y. et al. Site-Selective Atomic Layer Precision Thinning of MoS_2_ via Laser-Assisted Anisotropic Chemical Etching. *ACS Appl. Mater. Interfaces* **11**, 39385–39393 (2019).

39. Kang, S. et al. Phase-controllable laser thinning in MoTe_2_. *Appl. Surf. Sci.* **563**, (2021).

40. Castellanos-Gomez, A., Quereda, J., Van Der Meulen, H. P., Agraït, N. & Rubio-Bollinger, G. Spatially resolved optical absorption spectroscopy of single- and few-layer MoS_2_ by hyperspectral imaging. *Nanotechnology* **27**, (2016).

41. Jin, Q., Liu, N., Chen, B. & Mei, D. Mechanisms of Semiconducting 2H to Metallic 1T Phase Transition in Two-dimensional MoS_2_ Nanosheets. *J. Phys. Chem. C* **122**, 28215–28224 (2018).

42. Pető, J. et al. Moderate strain induced indirect bandgap and conduction electrons in MoS_2_ single layers. *npj 2D Mater. Appl.* **3**, 1–6 (2019).

43. Conley, H. J. et al. Bandgap engineering of strained monolayer and bilayer MoS_2_. *Nano Lett.* **13**, 3626–3630 (2013).

44. Mouri, S., Miyauchi, Y. & Matsuda, K. Tunable photoluminescence of monolayer MoS_2_ via chemical doping. *Nano Lett.* **13**, 5944–5948 (2013).

45. Lien, D. H. et al. Electrical suppression of all nonradiative recombination pathways in monolayer semiconductors. *Science* **364**, 468–471 (2019).

46. Wang, H. et al. Electrochemical tuning of vertically aligned MoS_2_ nanofilms and its application in improving hydrogen evolution reaction. *Proc. Natl. Acad. Sci.* **110**, 19701–19706 (2013).

47. Pandey, M., Bothra, P. & Pati, S. K. Phase Transition of MoS_2_ Bilayer Structures. *J. Phys. Chem. C* **120**, 3776–3780 (2016).

48. Cho, S. et al. Phase patterning for ohmic homojunction contact in MoTe_2_. *Science* **349** (2015).

49. Lopez-Sanchez, O., Lembke, D., Kayci, M., Radenovic, A. & Kis, A. Ultrasensitive photodetectors based on monolayer MoS_2_. *Nat. Nanotechnol*. **8**, 497–501 (2013).

50. Zhang, W. et al. High-gain phototransistors based on a CVD MoS_2_ monolayer. *Adv. Mater.* **25**, 3456–3461 (2013).

51. Klots, A. R. et al. Probing excitonic states in suspended two-dimensional semiconductors by photocurrent spectroscopy. *Sci. Rep.* **4**, 1–7 (2014).

52. Wang, W. et al. Photoresponse-Bias Modulation of a High-Performance MoS_2_ Photodetector with a Unique Vertically Stacked 2H-MoS_2_/1T@2H- MoS_2_ Structure. *ACS Appl. Mater. Interfaces* **12**, 33325–33335 (2020).

53. Yin, Z. et al. Single-layer MoS_2_ phototransistors. *ACS Nano* **6**, 74–80 (2012).

54. Kaushik, N. et al. Schottky barrier heights for Au and Pd contacts to MoS_2_. *Appl. Phys. Lett.* **105**, (2014).

55. Chee, S. S. et al. Lowering the Schottky Barrier Height by Graphene/Ag Electrodes for High-Mobility MoS_2_ Field-Effect Transistors. *Adv. Mater.* **31**, 1–7 (2019).

56. Wang, J. et al. High Mobility MoS_2_ Transistor with Low Schottky Barrier Contact by Using Atomic Thick h-BN as a Tunneling Layer. *Adv. Mater.* **28**, 8302–8308 (2016).

57. Goh, K. E. J. et al. Quantum transport in two-dimensional WS_2_ with high-efficiency carrier injection through indium alloy contacts. *ACS Nano* **14**, 13700–13708 (2020).

58. Ngo, T. D. et al. Control of the Schottky Barrier and Contact Resistance at Metal–WSe_2_ Interfaces by Polymeric Doping. *Adv. Electron. Mater.* **6**, 1–7 (2020).

59. Pandey, S. et al. Transition from direct to Fowler-Nordheim tunneling in chemically reduced graphene oxide film. *Nanoscale* **6**, 3410–3417 (2014).

60. Lee, S. Y. et al. Large Work Function Modulation of Monolayer MoS_2_ by Ambient Gases. *ACS Nano* **10**, 6100–6107 (2016).

61. Nourbakhsh, A. et al. MoS_2_ Field-Effect Transistor with Sub-10 nm Channel Length. *Nano Lett.* **16**, 7798–7806 (2016).

62. Lai, Z. et al. Metastable 1T′-phase group VIB transition metal dichalcogenide crystals. *Nat. Mater.* **20**, 1113–1120 (2021).

63. Pierucci, D. et al. Evidence for a narrow band gap phase in 1T′ WS_2_ nanosheet. *Appl. Phys. Lett.* **115**, (2019).

64. Liu, Q. et al. Stable Metallic 1T-WS_2_ Nanoribbons Intercalated with Ammonia Ions: The Correlation between Structure and Electrical/Optical Properties. *Adv. Mater.* **27**, 4837–4844 (2015).

65. Ling, M. et al. Phase-Controllable Synthesis of Multifunctional 1T-MoSe_2_ Nanostructures: Applications in Lithium-Ion Batteries, Electrocatalytic Hydrogen Evolution, and the Hydrogenation Reaction. *ChemElectroChem* **8**, 4148–4155 (2021).

66. Hanson, E. D. et al. Phase engineering and optical properties of 2D MoSe_2_ : Promise and pitfalls. *Mater. Chem. Phys.* **225**, 219–226 (2019).

67. Sokolikova, M. S., Sherrell, P. C., Palczynski, P., Bemmer, V. L. & Mattevi, C. Direct solution-phase synthesis of 1T’ WSe_2_ nanosheets. *Nat. Commun.* **10**, (2019).

68. Friend, R. H. & Yoffe, A. D. Electronic properties of intercalation complexes of the transition metal dichalcogenides. *Adv. Phys.* **36**, 1–94 (1987).

69. Huang, Y. et al. Chemical prelithiation of Al for use as an ambient air compatible and polysulfide resistant anode for Li-ion/S batteries. *J. Mater. Chem. A* **8**, 18715–18720 (2020).

70. Zhu, X. et al. Exfoliation of MoS_2_ Nanosheets Enabled by a Redox-Potential-Matched Chemical Lithiation Reaction. *Nano Lett.* **22**, 2956–2963 (2022).

71. Zhang, X. et al. An electrode-level prelithiation of SiO anodes with organolithium compounds for lithium-ion batteries. *J. Power Sources* **478**, 229067 (2020).

72. Li, F., Cao, Y., Wu, W., Wang, G. & Qu, D. Prelithiation Bridges the Gap for Developing Next-Generation Lithium-Ion Batteries/Capacitors. *Small Methods* **6**, 1–23 (2022).

73. Wang, G. et al. High performance lithium-ion and lithium–sulfur batteries using prelithiated phosphorus/carbon composite anode. *Energy Storage Mater*. **24**, 147–152 (2020).
